# Significant chlorine emissions from biomass burning affect the long-term atmospheric chemistry in Asia

**DOI:** 10.1093/nsr/nwae285

**Published:** 2024-08-16

**Authors:** Di Chang, Qinyi Li, Zhe Wang, Jianing Dai, Xiao Fu, Jia Guo, Lei Zhu, Dongchuan Pu, Carlos A Cuevas, Rafael P Fernandez, Weigang Wang, Maofa Ge, Jimmy C H Fung, Alexis K H Lau, Claire Granier, Guy Brasseur, Andrea Pozzer, Alfonso Saiz-Lopez, Yu Song, Tao Wang

**Affiliations:** Atmospheric Chemistry Department, Max Planck Institute for Chemistry, Mainz 55128, Germany; Department of Atmospheric Chemistry and Climate, Institute of Physical Chemistry Blas Cabrera, CSIC, Madrid 28006, Spain; Department of Civil and Environmental Engineering, The Hong Kong Polytechnic University, Hong Kong 999077, China; Environment Research Institute, Shandong University, Qingdao 266000, China; Division of Environment and Sustainability, The Hong Kong University of Science and Technology, Hong Kong 999077, China; Department of Civil and Environmental Engineering, The Hong Kong Polytechnic University, Hong Kong 999077, China; Environmental Modeling Group, Max Planck Institute for Meteorology, Hamburg 20146, Germany; Department of Civil and Environmental Engineering, The Hong Kong Polytechnic University, Hong Kong 999077, China; Institute of Environment and Ecology, Tsinghua Shenzhen International Graduate School, Tsinghua University, Shenzhen 518000, China; Environmental Central Facility, Institute for the Environment, The Hong Kong University of Science and Technology, Hong Kong 999077, China; Atmospheric Chemistry Modeling & Remote Sensing Research Group, Southern University of Science and Technology, Shenzhen 518055, China; Atmospheric Chemistry Modeling & Remote Sensing Research Group, Southern University of Science and Technology, Shenzhen 518055, China; Department of Atmospheric Chemistry and Climate, Institute of Physical Chemistry Blas Cabrera, CSIC, Madrid 28006, Spain; Institute for Interdisciplinary Science (ICB), National Research Council (CONICET), FCEN-UNCuyo, Mendoza 5501, Argentina; Institute of Chemistry, Chinese Academy of Sciences, Beijing 100190, China; Institute of Chemistry, Chinese Academy of Sciences, Beijing 100190, China; Division of Environment and Sustainability, The Hong Kong University of Science and Technology, Hong Kong 999077, China; Division of Environment and Sustainability, The Hong Kong University of Science and Technology, Hong Kong 999077, China; NOAA Chemical Sciences Laboratory/CIRES, University of Colorado, Boulder, CO 80305, USA; Laboratoire d'Aerologie, CNRS, University of Toulouse UPS, Toulouse 31062, France; Department of Civil and Environmental Engineering, The Hong Kong Polytechnic University, Hong Kong 999077, China; Environmental Modeling Group, Max Planck Institute for Meteorology, Hamburg 20146, Germany; Atmospheric Chemistry Observation & Modeling Laboratory, National Center for Atmospheric Research, Boulder, CO 80305, USA; Atmospheric Chemistry Department, Max Planck Institute for Chemistry, Mainz 55128, Germany; Department of Atmospheric Chemistry and Climate, Institute of Physical Chemistry Blas Cabrera, CSIC, Madrid 28006, Spain; State Key Joint Laboratory of Environmental Simulation and Pollution Control, Department of Environmental Science, Peking University, Beijing 100871, China; Department of Civil and Environmental Engineering, The Hong Kong Polytechnic University, Hong Kong 999077, China

**Keywords:** biomass burning emission, chlorine emission inventory, tropospheric halogen chemistry, atmospheric oxidants, CAM-Chem model

## Abstract

Biomass burning (BB) is a major source of trace gases and particles in the atmosphere, influencing air quality, radiative balance, and climate. Previous studies have mainly focused on the BB emissions of carbon and nitrogen species with less attention on chlorine. Reactive chlorine chemistry has significant effects on atmospheric chemistry and air quality. However, quantitative information on chlorine emissions from BB, particularly the long-term trend and associated atmospheric impacts, is limited both on regional and global scales. Here, we report a long-term (2001–2018) high-resolution BB emission inventory for the major chlorine-containing compounds (HCl, chloride, and CH_3_Cl) in Asia based on satellite observations. We estimate an average of 730 Gg yr^−1^ chlorine emitted from BB activity in Asia, with China contributing the largest share at 24.2% (177 Gg yr^−1^), followed by Myanmar at 18.7% and India at 18.3%. Distinct seasonal patterns and significant spatial and interannual variability are observed, mainly driven by human-mediated changes in agricultural activity. By incorporating the newly developed chlorine emission inventory into a global chemistry-climate model (CAM-Chem), we find that the BB-chlorine emissions lead to elevated levels of HCl and CH_3_Cl (monthly average up to 2062 and 1421 parts per trillion by volume (pptv), respectively), subsequently resulting in noticeable changes in oxidants (up to 3.1% in O_3_ and 17% in OH radicals). The results demonstrate that BB is not only a significant source of air pollutants but also of oxidants, suggesting a larger role of BB emissions in the atmospheric chemistry and oxidation process than previously appreciated. In light of the projected increase in BB activity toward the end of the century and the extensive control of anthropogenic emissions worldwide, the contribution of BB emissions may become fundamental to air quality composition in the future.

## Teaser

Biomass burning releases substantial chlorine-containing species, driving significant long-term and widespread impacts on atmospheric chemistry and oxidation in Asia, revealing a crucial and previously underappreciated role in air quality and climate interactions.

## INTRODUCTION

Atmospheric chlorine (Cl) chemistry can affect the chemical composition of the atmosphere through numerous reactions and processes. A growing body of observational and modelling studies has demonstrated that Cl chemistry significantly perturbs the atmospheric budgets of hydrocarbons, nitrogen, ozone, and greenhouse gases [1–5], therefore considerably affecting the air quality and climate. The chorine species actively contribute to atmospheric chemistry primarily by releasing chlorine atoms or radicals from the photodissociation and reactions of natural and anthropogenic reactive chlorinated species [6]. For example, the photochemical decay of CH_3_Cl in the stratosphere releases Cl and ClO, which catalyze the ozone (O_3_) loss in the stratosphere [1]. In polluted troposphere, the photolysis of abundant Cl_2_, ClNO_2_, and HOCl can provide an important source of Cl radicals, and enhance atmospheric oxidation capacity, volatile organic compounds (VOCs) oxidation, and O_3_ formation [6–8].

Sea-salt aerosols (SSA) have been suggested to be the dominant source of reactive Cl to the troposphere [9,10]. In early model simulations, global tropospheric chlorine mainly resulted from SSA, and most of the chlorine over continental regions was attributed to the long-range transport of SSA [11,12]. In recent inland field studies, significant levels of tropospheric chlorine species (1–2 parts per billion by volume (ppbv)) were observed and suggested to stem from anthropogenic sources other than sea salt [13]. Anthropogenic fossil fuel combustion and biomass burning (BB) have been suggested to be the main sources of atmospheric reactive Cl over the continent [9]. The efforts devoted to improving air quality have resulted in a reduction in the emissions from human-related activities, while BB is largely uncontrolled in most regions of the world, suggesting a persistent role of BB in the global Cl budget. This is consistent with many observation evidences which indicate the ubiquitous emissions of chlorinated species from open fires [14–17]. Moreover, climate change results in an increase in global surface temperature, and consequently an increase in the number of, and potential for, open fires [18].

With increasing awareness of the significance of chlorine in atmospheric chemistry, global and regional chemistry-climate models have incorporated some condensed chlorine chemistry mechanisms in the model [6]. Simulations have been performed to probe the effects of atmospheric chlorine on methane lifetime, tropospheric O_3_ formation and radical cycling [7,8,19]. Along with uncertainties in the model mechanisms and simplified assumptions, another primary area of uncertainty is the issue of the chlorine emission source driving the chemistry. The comprehensive effect of chlorine emitted from BB on air quality, however, has not been considered and investigated in previous studies. The BB emissions of greenhouse gases, particulate matter, and VOCs have been extensively reported (e.g. [20–26]), but little attention is paid to the BB Cl species [27–29], therefore limiting our knowledge of the full BB impact in the atmosphere.

The first global estimation of BB-Cl was the Reactive Chlorine Emissions Inventory (RCEI) with 1°×1° (∼100 km × 100 km) resolution, which was based on Cl-emission ratios referenced to carbon emissions or the fuel Cl content for the year 1990 [14]. Fu *et al*. [30] compiled an emission inventory of HCl and particulate chloride in China for 2014 with a 0.1°×0.1° (∼10 km × 10 km) resolution, in which BB was identified as the largest contributor (>50%) of total Cl emissions in China. In contrast, a more recent global emission inventory of HCl and particulate chloride for 1960–2014, which was developed based on the activity data and uniform emission factors, reported a very limited contribution of BB to the total Cl emissions, particularly in China [31]. Large discrepancies remain among these previous emission datasets. The contribution of BB in regional and global reactive Cl budget, and its variations under a changing climate have until now remained unknown. The effect of BB-Cl on tropospheric photochemistry, especially in Asia, remains largely unexplored, across different timescales.

## RESULTS AND DISCUSSION

### Trends of BB chlorine emissions in Asia

**Figure 1.**
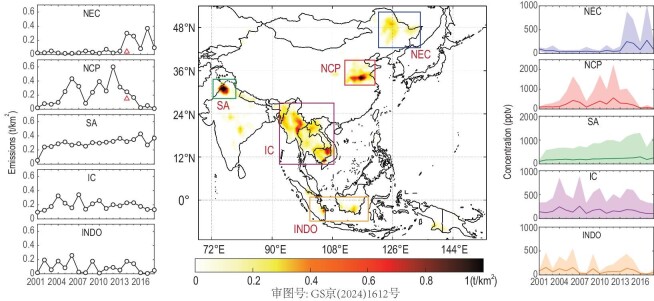
Spatial distributions and temporal variability of total Cl species (Cl^−^, HCl, CH_3_Cl) emitted from open biomass burning over the 2001–2018 period. The black lines in the left column show the calculated Cl emissions (sum of Cl^−^+HCl + CH_3_Cl) for the five selected regions, and the red triangles represent the anthropogenic emissions in 2014. The area plots in the right column display the time series of simulated total Cl concentrations attributed to open biomass burning. The solid lines represent average annual concentrations, with the shading indicating variability across all modelling grids within the respective region. NEC, Northeast China; NCP, North China Plain; SA, South Asia; IC, Indochina; INDO, Indonesia.

Motivated by the above, we combined satellite observations of burned areas with the emission factor (EF), fuel load (FL), and combustion factor (CF) to develop a long-term (from 2001 to 2018) and high-resolution (500 m) Cl emission inventory from open BB. The study region is shown in Fig. 1, one of the regions in Asia with substantial BB activity. Satellite data offers a great tool for estimating the Earth's surface emissions resulting from various activities, including BB, especially when detailed bottom-up statistics data and ground-based measurements are lacking. The updated version of the Moderate Resolution Imaging Spectrometer (MODIS) burned area product, MCD64A1 version 6 [32], was used to produce the 500 m resolution burned area maps. The land cover information was derived from the Level 3 MODIS Land Cover Type Product (MCD12Q1) [33], and the 17 vegetation classes were grouped into five broad types, namely forests, shrublands, grasslands, croplands, and others. The FL was determined from the total aboveground biomass (AGB) density, including the aboveground living biomass and litterfall for each ecosystem. The agricultural residue burning in the open field was also considered, which would be a large proportion (>90%) of the total BB in China's mainland. The EFs, which are strongly dependent on vegetation type, fuel size, fuel moisture, and combustion efficiency, were obtained from the vegetation-specific local measurements for the study regions during recent years [27,34–39]. More information on the inventory methodology is described in detail in the Methods and Supplementary Materials. The estimated Cl emission inventory in this work includes particulate chloride (Cl^−^), the major gas-phase Cl component (HCl) and organic chlorine (CH_3_Cl), which constituted most of the chlorine emitted from BB.

In Asia, the total emission of Cl-containing species (comprising particulate Cl^−^, gaseous HCl, and CH_3_Cl) from BB was estimated to be 730 Gg yr^−1^ on average, varying from 240 to 1083 Gg yr^−1^ during 2001–2018 (Table S1). Henceforth, all total Cl amounts reported in this study refer to the total mass or concentrations. China was the largest contributor to the total Cl emissions in Asia (24.2%), with annual average emissions of 177 Gg Cl yr^−1^, followed by Myanmar (18.7%), and India (18.3%), with average emissions of 137 and 134 Gg Cl yr^−1^, respectively. The spatial distribution of 18-year averaged Cl emissions from open burning is illustrated in Fig. 1. The BB-Cl emissions were mostly located in agricultural-intensive and forested areas, remarkably represented by five high-density regions, i.e. northeastern China (NEC), the North China Plain (NCP), South Asia (SA), Indochina (IC), and Indonesia (INDO). Overall, the emissions from the five regions accounted for an average of 81% of BB-Cl emissions in Asia during the last two decades. The most intensive area was found in SA with an average emission rate of 0.29 t_Cl_ km^−2^, followed by NCP (0.20 t km^−2^) and IC (0.19 t km^−2^).

The total Cl emissions from BB in Asia during 2001–2018 were substantially lower than that of 2526 Gg Cl yr^−1^ from RCEI, representative for the year 1990 (Table S2), which was estimated based on the emission ratios of Cl to CO_2_ and CO [14]. Two- to fourfold overestimations in both anthropogenic and BB Cl emissions in China were found for the RCEI compared to the updated emission inventory in 2014 [30]. The outdated and rough estimation without localized emission parameters hinders the use of RCEI in simulations for present-day conditions. Strict control policies on agricultural residue burning have been implemented in China during the last decade, especially in the NCP region, which has resulted in a significant reduction in BB emissions [40]. This can be seen in the trend of BB-Cl emissions on the NCP from 2001–2018 in Fig. 1. The Cl emissions over the NCP peaked with a value of 0.6 t km^−2^ in 2012 and declined rapidly by 90% to 0.01 t km^−2^ in 2018. However, the results from Zhang *et al*. [31] were approximately an order of magnitude lower compared to the present work (Table S2), which is associated with the different satellite products and the different EF parameters used in the calculation. The local measurement results of EFs used in this work, especially in China (Table S3), are much higher compared to the global uniform data used by Zhang *et al*. [31]. Moreover, while prior studies suggested that particulate Cl^−^ was predominant in total Cl emissions from BB, our present study reveals that gaseous HCl emissions are comparable but slightly lower than particulate Cl^−^ (Table S2).

**Figure 2.**
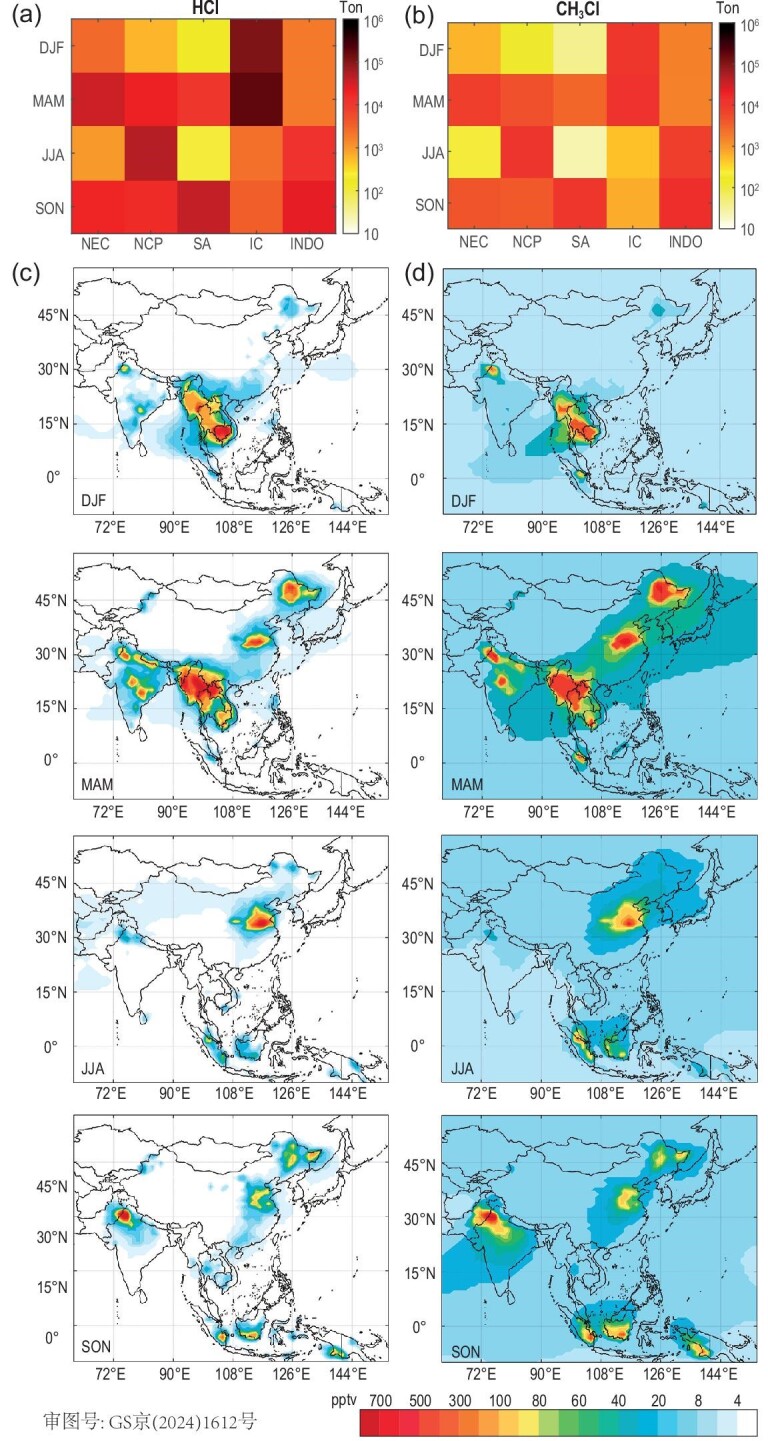
Seasonal variations of biomass burning (BB) Cl emissions and simulated Cl levels in Asia. (a) Emission intensity (tons) of inorganic Cl in different seasons and regions. (b) The same as (a) but for CH_3_Cl. (c) Seasonal variations in HCl mixing ratios (pptv) at the surface layer. (d) The same as (c) but for CH_3_Cl. Different seasons are represented as follows: December to February (DJF) for winter, March to May (MAM) for spring, June to August (JJA) for summer, and September to November (SON) for autumn.

In China, the in-field burning of crop residue was the largest contributor to total BB Cl emissions, accounting for an average fraction of 92%, while the open fires in forests, shrublands, and grasslands together contributed the remaining 8%. The estimated total inorganic Cl loading in China (281 Gg Cl yr^−1^ in 2014, Table S2) falls well within the previously reported ranges. A bottom-up estimation reported 511 Gg Cl yr^−1^ (in 2014) using government provincial-level statistics and survey burning data [30], while another study estimated only 45 Gg Cl yr^−1^ in the same year [31]. The geophysical distribution of BB-Cl emissions was associated with rural population densities and land-use patterns. The monthly and seasonal variations in BB emissions agree well with a statistical survey on the nationwide open burning of crop residues [41]. NEC and NCP served as the major agricultural regions and in-field crop residue combustion areas [42], contributing the most to BB-Cl emissions in China. The NCP had an uppermost contribution from 2005 to 2014 due to the residue burning of wheat, rice, and corn in late spring (Fig. 2 and Fig. S1). In contrast to the significant decline in BB on the NCP, a marked rising trend has emerged in the NEC region during the last five years, with a peak value of 0.37 t km^−2^ in 2017. The hot spots of BB in China gradually moved from the NCP to the NEC region during the last several years, and the peak month for the total emissions in China correspondingly changed from June to April. A similar upward trend for NEC was also noticed in previous works [43,44], and the variations were mainly associated with the planting structure adjustment related to agricultural policies, especially the increase in planting areas from 2013 to 2017, but insufficient enforcement of government control measures [43]. Compared to other anthropogenic (ANT) emissions (coal combustion, industrial processes, and solid waste incineration), the proportion of the BB contribution was significantly prominent in the total Cl emissions in these two regions, with a BB:ANT ratio of 1.6:1 on the NCP and 7.5:1 in NEC in 2014 (Fig. S2). The emission and contributions from BB, as well as the subsequent impacts on air quality, will be more significant in NEC in the future assuming a relatively steady or decreasing anthropogenic emission but increasing BB activities. Therefore, stricter and targeted pollution control strategies are needed.

The BB-Cl emissions in other Asian regions, e.g. SA, IC and INDO, exhibited smaller fluctuations over the past two decades, as depicted in Fig. 1. This trend is consistent with the trend of BB activities reported in previous research [31,45]. The total Cl emission in India was 134 Gg yr^−1^ (varied from the lowest of 32 Gg in 2001 to the highest of 187 Gg in 2009), which is ∼10 times larger than the results presented by Zhang *et al*. [31]. The spatial distributions of BB-Cl in India are generally in agreement with previous observations on BB activities, with a dominant contribution from the SA region (including Punjab and Haryana) [46]. BB-Cl from the SA region displayed a steadily increasing trend, which was mainly attributed to increasing agricultural (rice crop) residue burning [47] in the post-monsoon season (October–November, Fig. S1). It is in line with the high chlorine levels observed in the post-monsoon period in this region [48]. Previous studies have suggested that these two Indian states contributed more than 90% of the post-monsoon fire intensity in India, which coincides with the unfavourable meteorological conditions and leads to severe air quality degradation and public health consequences across the densely populated Indo-Gangetic Plain [49,50]. Surface burning in the forest area played a major role in Cl emissions in the IC region (Fig. S1), which was quite stable in the last two decades (Fig. 1). The peak BB emission was found in the dry season, i.e. December–May (Fig. S1), a pattern consistent with statistical data on forest fires gathered by the Forest Fire Control Division National Park. Most fires in the IC region (e.g. Thailand, Myanmar) are human-initiated (e.g. the gathering of forest non-timber products, hunting, agricultural residue burning for land clearing, incendiary fires, etc.), especially by rural residents living in nearby forested areas [51–55]. The Cl emissions in the INDO region were related to peat burning in forest/shrubland areas with high carbon density (Fig. S1, [45]). The peak emissions were mostly found from September to October (Fig. 2 and Fig. S1), and the drought conditions during the El Niño years (e.g. 2006 and 2015) enhanced the fire occurrence and Cl emissions. The burning extent of carbon‐rich peatlands, fire-driven deforestation and agricultural management, combined with synoptic meteorology, were the main factors dominating the variation in BB emissions and exacerbating haze episodes over Equatorial Asia [56]. The peak season of BB emissions varies across different regions, influenced by a range of climatic and ecological factors as well as human activities such as agriculture. This variability underscores the diverse nature of BB occurrences worldwide and its impact on atmospheric processes.

Previous studies reported a significant underestimation of Cl based on the existing emission inventory in comparison with the observations [57,58]. The Cl radicals activated from Cl emissions exert a strong influence on tropospheric oxidation and radical cycling, but are poorly simulated in atmospheric chemistry models [57–59]. Therefore, understanding the sources and evolution of Cl emissions is crucial for constraining the impacts of Cl-containing species on a regional or global scale.

### Impacts on atmospheric chlorine levels

We have incorporated the developed BB-Cl emission inventory into a global chemistry-climate model, CAM-Chem with comprehensive reactive halogen (including Cl) chemistry. We conducted four simulation scenarios, noBB (without neither BB nor ANT; 2001 to 2018), BB (with BB; without ANT; 2001–2018), noBB_ANT (without BB; with ANT; 2014), and BB_ANT (with both BB and ANT; 2014) (Table S4), and evaluated the BB-Cl effects on reactive Cl abundance and oxidation capacity. For the configuration with ANT, the anthropogenic emissions of chlorine came from Fu *et al*. [30], which were derived from the detailed county-level activity data and corresponding emission factors. The model simulation performance was first validated against ground-based and satellite-derived observation data (Table S5, Figs S3–S5), which suggested that the simulation results were generally in line with the observations and that the inclusion of BB-Cl helped reproduce the observed magnitude of Cl species.

The interannual variations in the simulated mixing ratios of Cl species (Fig. 1) are consistent with the emission trends in the five hotspot regions. The highest total Cl of 2200 parts per trillion by volume (pptv, monthly average) was simulated for the NCP in 2012, and since then, the Cl levels on the NCP have been decreasing in recent years. The contribution of BB-Cl to the atmospheric Cl abundance in China in 2014 (the year with available anthropogenic Cl emissions) was ∼54% of the China national average, with a value >70% in some urban areas, and up to 100% in some rural and forested areas (Fig. S2). BB emissions also persistently contributed 800 − 1000 pptv of atmospheric Cl in the SA and IC regions during the last two decades. The spatial distributions of the modelled Cl in India are generally in agreement with the measurements, with a high concentration in Dehli [46]. Up to 700 ppt of ClNO_2_ were observed in an urban background site in New Delhi in Jan–Feb 2019 [60], and this is consistent with the hot spot shown in Fig. 2. In the SA region, the co-condensation process of HCl with water on aerosols has been reported to result in a 50%−70% visibility reduction during winter [46,61]. Previous observations captured significant chloride fractions in the atmospheric particles, and their sources are complicated. A comparison of the simulations to observations suggests that if BB-Cl is not considered, one might leave out a noticeable fraction of the tropospheric Cl as indicated in previous studies [61,62] as well as Table S5 of the present study.

We also examined the seasonal variations in the simulated abundance of Cl (Fig. 2). The simulated HCl and CH_3_Cl shared similar seasonal and spatial variations (Fig. 2), although HCl was more confined near the emission region because of the scavenging effect while CH_3_Cl was more widespread due to its longer lifetime. The most significant effect was found in the spring season (i.e. March–May, MAM), with >1000 pptv of Cl simulated in vast areas of Asia, while SA, INDO, and IC experienced greater effects in fall and winter (Fig. 2). The average simulated concentrations of CH_3_Cl in China due to BB emissions ranged from 50 pptv during December to February (DJF) to the 358 pptv during March to May (MAM), which is ∼5% to 30% of the atmospheric mixing ratios measured in various Chinese cities (average of 952 ± 273 pptv, with a range of 651 ± 11 to 2008 ± 1068) [63] and in the Pearl River Delta region (249−256 pptv) [64]. The simulated CH_3_Cl concentrations in SA (Fig. 2d) were generally consistent with the observation results in spring (∼650 pptv), with slight underestimation [65]. BB CH_3_Cl can reach the free troposphere and stratosphere (Fig. S6), which partly explains the significant Cl species observed in the stratosphere [66].

Moreover, CAM-Chem simulations in Asia revealed that the inorganic chlorine contributed by sea-salt is typically at 1 to 10 μg/m^3^ over oceanic regions, ∼1 μg/m^3^ along the coast, and ∼0.1 μg/m^3^ in inland areas. The inclusion of BB-Cl emissions induced an increase of more than 0.1 (up to 1.0) μg/m^3^ in simulated inorganic chlorine levels in the key regions (NEC, NCP, SA, IC, and INDO), corresponding to an ∼100% increase in the core regions (and >40% in larger regions) compared to contributions solely from sea-salt aerosols (Fig. S7). It's worth noting that when averaged across the entire study domain (land and ocean) at the surface level, sea-salt contribution amounts to ∼3.50 μg/m^3^, while the contribution of BB-Cl is only ∼0.01 μg/m^3^.

### Impacts on atmospheric oxidants

**Figure 3.**
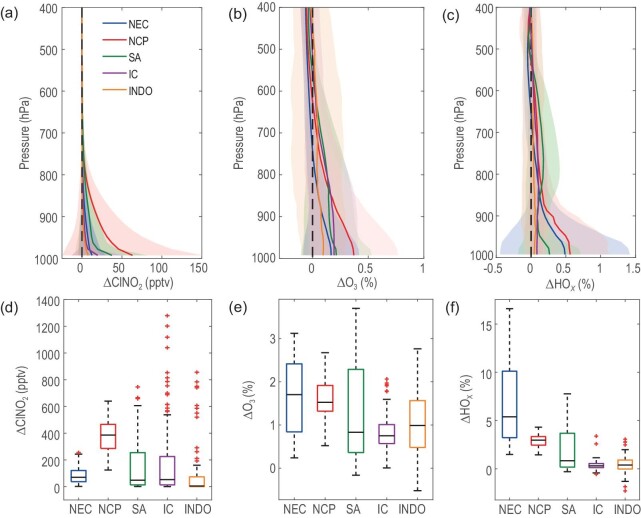
Simulated impacts of biomass burning (BB) Cl on ClNO_2_, O_3_, and HO*_x_*. The upper panel demonstrates the vertical patterns of the change in ClNO_2_ (a), O_3_ (b), and HO*_x_* (c) attributed to BB Cl averaged over the 18 (2001–2018) simulated years. The lower panel illustrates the maximum influence of BB Cl on ClNO_2_ (d), O_3_ (e), and HO*_x_* (f) at the ground surface. The box-and-whisker plots show the variations in the change for each species across the modelling domain for different regions (medians are presented as horizontal bars, boxes show the first and third quartiles, and whiskers show the 10th and 90th percentiles). The ClNO_2_ concentrations were derived from the midnight simulation results, while the O_3_ and HO*_x_* concentrations were noontime simulation results.

We assessed the influences of BB-Cl on atmospheric oxidants and radical precursors (Fig. 3). Chlorine has been reported to increase the oxidation capacity in polluted regions, particularly through conversion to a reactive form such as ClNO_2_, which is formed from the heterogeneous reactions of N_2_O_5_ with aerosol chloride or N_2_O_5_ with HCl in the presence of other aerosol surfaces [67–69]. Our results show that considering BB-Cl emissions, the simulated 18-yr average of ClNO_2_ would increase by 10 to 60 pptv over the study regions, with a maximum of 1200 pptv (monthly concentration) found at the surface in IC (Fig. 3). The influence of BB Cl on ClNO_2_ could reach the free troposphere (800 hPa) but would be mostly within the planetary boundary layer (900 hPa). The Asian summer/winter monsoon and summertime typhoons can efficiently transport air pollutants in Asia to the upper troposphere and lower stratosphere [70–73]. The mixing ratios of ClNO_2_ in each region (Fig. S8) showed a large seasonal variation following the trend of BB Cl emissions (Fig. 2). The impacts were more significant for the region with high NO*_x_* emissions, i.e. the NCP. The 90th-percentile monthly mixing ratios of ClNO_2_ were simulated to be 243, 640, 605, 537, and 160 pptv for NEC, NCP, SA, IC, and INDO regions, respectively (Fig. 3d). These simulated ClNO_2_ mixing ratios induced by BB-Cl emissions are quite comparable to the field-observed ambient ClNO_2_ levels (contributed by various Cl sources) in North America, Europe, and Asia, ranging between several and hundreds of pptv [13,68,74], highlighting the potential significance of BB Cl from Asia. It is noteworthy that the simulated ClNO_2_ was elevated in the SA and IC regions, but no such measurements are available, calling for more field studies to understand BB-Cl impacts.

The resulting influence of BB Cl on surface O_3_ (Fig. 3b; Fig. S9) was predicted to be mild, with increases of 1.63% ± 0.87%, 1.56% ± 0.43%, 1.38% ± 1.18%, 0.81% ± 0.39%, and 1.03% ± 0.79% in the NEC, NCP, SA, IC, and INDO regions, respectively. In contrast, the effects tended to be negative for O_3_ in the free troposphere (Fig. 3b) and stratosphere (Fig. S6). The surface impact is dominated by inorganic Cl reaction and the latter is dominated by CH_3_Cl photodecomposition. That is why the former produces ozone and the latter reduces it. During the months with intensive BB activities (and elevated ClNO_2_), BB Cl resulted in a much more noticeable effect on O_3_ (Fig. 3e). Interestingly, the largest change in O_3_ was found in SA, while the largest monthly ClNO_2_ was found in IC, implying the nonlinear effect of Cl chemistry on O_3_ formation. The BB Cl effect on HO*_x_* (OH and HO_2_; Fig. 3c; Fig. S10) had a similar pattern to that of O_3_ but with larger values. Surprisingly, the largest monthly change in OH reached 17% in NEC in November 2015 (Fig. 3f). For other regions, the maximum increase effects were 2.89% ± 0.77%, 2.01% ± 2.40%, 0.36% ± 0.44%, and 0.49% ± 0.95% in the NCP, SA, IC, and INDO regions, respectively. The present study aims to quantify the long-term impacts of BB chlorine on air quality in Asia. However, it's important to note that BB emission of CH_3_Cl, a long-lived chlorine species with an atmospheric lifetime of ∼300 days [75], also affects the upper tropospheric and lower stratospheric ozone abundance. As shown in Fig. S6, the average levels of CH_3_Cl due to BB above 400 hPa across the main Asian regions are ∼20 pptv, which resulted in O_3_ mixing ratio changes of approximately −0.25% at these altitudes.

We then examined the possible cause for the disparate responses of oxidants to BB Cl emission (Fig. S11). We found that the effects of BB Cl on O_3_ were larger in the regions with higher NO*_x_* and VOC concentrations, which explains the relatively smaller increase in O_3_ in the IC region even though it had the highest ClNO_2_. We also noted that the relative change in OH had a strong correlation with the relative humidity due to the required presence of water vapor in producing OH radicals initiated by O_3_ photolysis (R11–R12). A negative correlation was found between OH changes and temperature, implying that the enhancing effect of Cl on OH was more significant in cold environments (with lower radiation and lower OH levels). This connection highlights the importance of the peak seasons of BB activity in affecting atmospheric chemistry and oxidation capacity (Fig. 2). During the cold season in the Northern Hemisphere, enhanced anthropogenic emissions and unfavourable meteorological conditions (e.g. higher humidity) contribute to the accumulation of pollutants, and the BB-Cl induced enhancements in oxidation capacity further promote the photochemistry and the formation of secondary pollution.

## DISCUSSION AND OUTLOOK

Ongoing climate simulations have forecasted the potential range of BB activities under different shared socio-economic pathway (SSP) scenarios in the future [76]. Climate warming and drying will lead to more severe and frequent forest fires in most regions. Here, we project the trend and potential ranges of BB Cl emissions referenced to the reported emission intensity of BB black carbon and CO in the five key regions toward the end of the century for the SSP1-2.6, SSP2-4.5, and SSP5-8.5 scenarios (Methods; Fig. S12). The projected BB Cl under SSP1-2.6 and SSP2-4.5 would generally remain at the present level, while that under the SSP5-8.5 scenario would significantly increase until 2100, particularly in SA and on the NCP. Anthropogenic chlorine emissions are closely related to coal-burning activities, which are characterized by sulfur emissions. While BB emissions are projected to decrease or increase in various Asian regions, anthropogenic sulfur emissions are mostly expected to decrease, albeit to varying extents depending on the scenarios [77]. Consequently, a decrease in future anthropogenic chlorine sources is anticipated. This trend has important implications for the significance of BB (including chlorine and other emitted species), particularly given the ongoing warming of the global climate and the increased likelihood and extent of wildfires.

The warming climate is transforming landscapes and causing more frequent forest fires and wildfires in both the Northern and Southern hemispheres [78]. Forest fires with unprecedented size and intensity were experienced in eastern Australia in 2019–2020 [79], in the western USA during 2015–2020 [80], and in the Amazon [81]. BB is traditionally considered a significant source of air pollutants and greenhouse gases. This work demonstrates that BB also supplies Cl species that perturb the oxidation capacity both directly (via Cl) and indirectly (via HO*_x_* and O_3_). Unlike air pollutants (e.g. black carbon, NO*_x_*, etc.) and greenhouse gases (e.g. CO_2_) from BB that have been intensively studied for large fires (e.g. [82]), BB-Cl species emissions and their atmospheric (tropospheric and stratospheric) impacts are less studied (e.g. [83]) and remain poorly quantified. Future *in-situ* observations are desired to further refine our understanding of BB-derived Cl sources. Furthermore, global warming tends to expand the fire-vulnerable areas and even shift toward extreme northern regions, e.g. Alaska and the Arctic area [84,85], by both nature and anthropogenic disturbances (land-use alteration and driven by agriculture) [86]. As we report for the NEC region, BB-Cl emissions could have more profound effects on atmospheric oxidation chemistry in cold environments/regions. The predicted increasing fire activities thus will have greater impacts on regional air quality and the ecosystem by releasing more reactive species or precursors. Failure to account for BB-Cl when formulating air pollution mitigation policies could lead to an underestimation of the required reductions in anthropogenic sources of air pollutants to achieve air quality targets. Further investigations are required to identify and quantify the Cl emissions from BB in different regions as a key to understanding the integrated roles of BB in shaping atmospheric chemistry, air pollution, and climate. We also suggest that future studies on BB should include Cl species to fully understand the impacts of BB on atmospheric chemistry.

## Supplementary Material

nwae285_Supplemental_File
