# Supplementary material for: Significant chlorine emissions from biomass burning affect the long-term atmospheric chemistry in Asia
Source: Natl Sci Rev. 2024 Aug 16;11(9):nwae285. doi: 10.1093/nsr/nwae285 (PMC11413532; doi:10.1093/nsr/nwae285)
Supplement: nwae285_Supplemental_File [file nwae285_supplemental_file.pdf]

Supplementary Materials for

Significant chlorine emissions from biomass burning affect the long-term atmospheric chemistry in Asia

Di Chang,[1#] Qinyi Li,[2,3,4#], Zhe Wang,[5]* Jianing Dai,[3,6] Xiao Fu,[3,7] Jia Guo,[8] Lei Zhu,[9] Dongchuan Pu,[9] Carlos A. Cuevas,[2] Rafael P. Fernandez,[10] Weigang Wang,[11] Maofa Ge,[11] Jimmy C.H. Fung,[5] Alexis K.H. Lau,[5] Claire Granier,[12,13] Guy Brasseur,[3,6,14] Andrea Pozzer,[1] Alfonso Saiz-Lopez,[2] Yu Song,[15] Tao Wang[3]

* Corresponding author: Zhe Wang (z.wang@ust.hk)

This PDF file includes:

Materials and Methods
Figs. S1 to S13
Tables S1 to S6
SI References

Materials and Methods

1. Methods and Data

The emissions from open biomass burning for specific species are estimated as a product of the burned area (BA), fuel load (FL), combustion factor (CF), and emission factor (EF) associated with different land cover types [1]. The land cover information required to characterize the emissions was derived from the Level 3 MODIS Land Cover Type Product (MCD12Q1), which provides a suite of science datasets (SDSs) at a 500-meter spatial resolution for each variable spanning from 2001to 2017 [2]. We substituted the 2017 MCD12Q1 data for the year 2018 and beyond. The 17 vegetation classes in the MCD12Q1 product were grouped into five broad types: forests (including evergreen needleleaf, evergreen broadleaf, deciduous needleleaf and deciduous broadleaf forests), shrublands (including closed and open shrublands, woody savannas and savannas), grasslands, croplands, and others (including wetlands, urban lands, barren land, water bodies, snow and ice) for further discussion.

1.1 Burned area (BA)

The new version of the Moderate Resolution Imaging Spectrometer (MODIS) burned area product, known as C6 MCD64A1 (accessible via https://e4ftl01.cr.usgs.gov/MOTA/MCD64A1.006/), was used to generate the burned area maps. C6 MCD64Al has a 500-m spatial resolution and a monthly temporal resolution, allowing for precise detection of burned areas at a 500 m pixel resolution. This product is generated based on a hybrid algorithm that employs MODIS version 6 surface reflectance imagery coupled with active fire input data. Since late 2016, improvements have been introduced to the algorithms in the MODIS burned area product Collection 5.1 (C5.1) MCD64A1 and MCD45A1, which facilitate better detection of smaller burns (ranging from 21 to 100 ha), modestly reduce the burn-date temporal uncertainty, and largely diminish the extent of unmapped areas [3]. Compared to the previous version, the C6 product detected a considerably larger burned area worldwide (26%), especially in cropland across Central and Southeast Asia. The satellite data are highly reliable for quantifying biomass burning across extensive landscapes, such as forest fires, savanna and grassland burning, but their capability to detect small-scale fire activities, such as agricultural burning, remains limited [4, 5]. To address this limitation, *Zhu* et al. [5] suggested some correction factors tailored to various vegetation types based on a reference fire product with a high resolution better than 30 m. These correction factors were applied as follows: 7.70 (5.9−8.2) for croplands, 0.80 (0.23−1.0) for mixed forests, 1.40 (0.98−2.7) for deciduous forests, 0.99 (0.69−1.1) for grasslands, and 0.95 for shrublands. In our study, we incorporated these correction factors into the BB data calculation for China, where small-scale open agricultural burning is significant. In addition, the MCD12Q1 land cover product was used to preprocess the burned area data to remove the grids that are designated as burned in the MCD64A1 product but were classified as urban, barren, water bodies or non-vegetated areas in the MCD12Q1 product.

1.2 Fuel loading (FL)

The total aboveground biomass (AGB) density for each grid includes the aboveground living biomass and litterfall within each ecosystem. The living biomass densities for large countries, i.e., China and India, were on a provincial or state basis (Table S6). An updated wall-to-wall

map of forest AGB density at 1-km resolution for China [6] was used as the forest woody loading, which fully considered the geographical variation. On average, the AGB density across China was 12±6.1 kg m^{-2}, with generally larger values observed in the southern regions compared to northern regions. For shrublands and grassland, we adopted the provincial data for China from previous literature [7, 8], as listed in Table S6. Litterfall, a notable contributor to carbon storage especially in forest regions [9], and failing to account for litterfall combustion might result in a substantial underestimation of biomass burning emissions. For other Asia regions, country-level biomass density data were used (Table S6). Furthermore, in this work, the litterfall densities for different land cover types (Table S6) were derived from regional measurement data [9-11].

The crop residue availability for China was calculated according to the statistics of farm crop production at the provincial level and residue-to-production ratio [12]. The fuel load for a specific crop residue ($FL_{i,k}$) was estimated according to the following equation given by *L Yang* et al.[13]:

$$FL_{i,k} = P_{i,k} \times RF_{i,k}$$

$P_{i,k}$ is the annual crop yield per unit of different crop species k in province i, which can be derived from the China Statistical Yearbook [14], except for Hong Kong, Macao, and Taiwan. $RF_{i,k}$ represents the residue factors for different crop species k in province i, which are defined as residue-to-production ratios according to *Wang* et al. [15]. The fuel loadings of crop residue in other countries were derived based on statistical data [16-18].

1.3 Emission factors (EFs)

The emission factors (EFs), defined as the mass of emitted species per unit mass of dry matter burned, are strongly dependent on factors such as vegetation type, fuel size, fuel moisture, and combustion efficiency [19]. In the present study, we prioritized the measurement data specific to local regions in recent years, despite huge amounts of data across the world. EFs for particulate chloride (Cl^-) and CH_3Cl were mainly collected from vegetation-specific local measurement campaigns [20-23] and are summarized in Table S3. As comprehensive measurements for HCl emission in Asia were lacking, we adopted EFs for HCl from previous global research efforts [24], as documented in Table S3. In addition, considering that corn, rice and wheat constitute the predominant agricultural crops in Asia and are frequently subjected to open burning [13], EFs for the crop residue combustion were derived by averaging the available data for these three crops.

1.4 Combustion Factors (CFs)

The combustion factor (CF) is the fraction of burnt biomass in a fire, and it is influenced by various factors including fuel type, fire intensity and fuel moisture content. Herein, we adopted CF values for different land cover types based on recent improvements in vegetation parameterization [25]. Specifically, the CF for woody fuel with tree cover exceeding 30% was set as 0.3 [26], while 0.5, 0.85, and 0.4 were used for closed shrublands, open shrublands, and woody savannas, respectively [25]. For herbaceous fuels and fine litter, CFs were assumed to be 0.95 [25]. The CFs of farm crop residues (0.86) were determined based on the weighted average for rice (0.89), wheat (0.86), corn (0.92), beans (0.68), tubers (0.8), cotton (0.8), peanuts (0.8), and rapeseed (0.82) [27].

2. Validation and comparison with the bottom-up method

A previous study has pointed out that the 500 m MCD45A1 product often fails to detect small-scale field crop residue burning [28], which can be a significant contributor to total emissions. Therefore, for comparison, we also computed emissions from field crop residue burning based on the bottom-up method, which was often used for emission inventories in previous studies. The amount of residue burned in the field was calculated as a product of annual crop yield [14], residue-to-production ratio, field burning percentage, dry matter fraction, and burning efficiency. It's worth noting that this method relies on certain simplifying assumptions, such as assuming a constant amount of crop straw being burned in fields or a constant burning efficiency, which can yield vastly different results under different assumptions [27, 29]. In our analysis, we adopted the approach proposed by Li et al. [27], which considered agriculture mechanization ratios to enhance the accuracy of the related activity data required for emission calculation.

To validate the accuracy of our burned area data, we compared the forest-burned areas for each province in China derived from the MCD64A1 product with national statistics on fire-affected forest area from 2004 to 2016 [14] (Fig. S12a). Both datasets showed that Heilongjiang was the largest contributor to the total forest burned area, followed by Inner Mongolia and Yunnan. As shown in Fig. S12a, the burned area derived from the MCD64A1 product is somewhat lower than the statistical data during 2004−2006. However, from 2007 to 2016, the forest burned areas derived from MCD64A1 were either comparable to or larger than the statistical results (Fig. S12a). On the provincial level, the MCD64A1 burned areas aligned well with statistics for southern China, including provinces like Sichuan, Anhui, Jiangxi, Fujian, Guangxi, and Hainan. Nevertheless, there was moderate underestimation by MCD64A1 for most northern regions, such as Hebei, Shandong, Shanxi, and Gansu. In Northeast China and Inner Mongolia, MCD64A1 notably underreported the burned area during 2004−2006 but slightly overpredicted the burned area after 2007.

The chlorine emissions derived from the bottom-up method and MCD64A1 product are compared in Fig. S12c, which shows good agreement in 2005, 2009, 2011 and 2013. However, there was no year-to-year variation observed in the bottom-up estimation, as it exhibited a linear relationship with the annual farm crop yield, which increased steadily. This trend was unexpected in view of the existence of control policies on crop residue burning [30, 31]. In contrast, noticeable annual fluctuations existed for the emissions derived from MCD64A1 product. The gradual increase over the period of 2001−2006 agreed well with many previous studies focused on $PM_{2.5}$ concentrations [31]. A significant reduction occurred in 2008 in response to the implementation of various burning control policies, and this inflection point in 2008 has also been reported in prior research [31].

Potential sources of uncertainty in the emission inventories include activity data, variability in land cover and land use changes, emission factors, combustion conditions, biomass characteristics, among others. To mitigate these uncertainties, additional local measurements of biomass density, characteristics, and emission factors for specific vegetation type are required.

3 CAM-Chem model and reactive chlorine chemistry mechanism

The CAM-Chem model is a global chemistry-climate model [32] and is the atmospheric component of the Community Earth System Model (CESM; [33]). The CAM-Chem model

includes comprehensive reactive halogen (chlorine, bromine, and iodine) chemistry, and detailed descriptions can be found elsewhere [34-38]. Here, we briefly introduce the reactive chlorine chemistry considered in the CAM-Chem model and its influence on key tropospheric oxidants (e.g., OH and O_3).

Chlorine species significantly perturb tropospheric chemistry [39, 40]. Reactive chlorine species can be emitted into the atmosphere from anthropogenic, biomass-burning, and natural sources in the form of chloride in aerosols and different gaseous species like HCl and CH_3Cl. The heterogeneous uptake of HNO_3 on sea-salt aerosols (SSA) will cause subsequent activation of chlorine from the aerosol phase to the gas phase (e.g., R1). The heterogeneous reaction of N_2O_5 on chloride-containing aerosol leads to the production of $ClNO_2$ (R2); the heterogeneous reaction of N_2O_5 with HCl in the presence of other aerosol surfaces could also result in the formation of $ClNO_2$ (R2) [41]. Chlorine chemistry starts with the photolysis of many chlorine-containing species to release chlorine atoms (e.g., R3-R4). Subsequently, chlorine atom reacts with VOCs to form secondary oxidants (e.g., OH, HO_2, and O_3) in the presence of NO_x (R5-9). In clean environments with low NO_x and VOC levels, chlorine atoms primarily consume O_3 and reduce OH levels (R10-12), although the reaction between ClO and HO_2 compensates for OH loss (R13-R14).

$$HNO_3 + SSA \rightarrow HCl \quad \text{(R1)}$$
$$N_2O_5 + SSA/HCl \rightarrow ClNO_2 + HNO_3 \quad \text{(R2)}$$
$$ClNO_2 + h\nu \rightarrow Cl + NO_2 \quad \text{(R3)}$$
$$CH_2Cl_2 + h\nu \rightarrow 2Cl \quad \text{(R4)}$$
$$Cl + VOC \rightarrow RO_2 + HCl \quad \text{(R5)}$$
$$RO_2 + NO \rightarrow NO_2 + RO \quad \text{(R6)}$$
$$HO_2 + NO \rightarrow NO_2 + OH \quad \text{(R7)}$$
$$NO_2 + hv \rightarrow NO + O(^3P) \quad \text{(R8)}$$
$$O(^3P) + O_2 \rightarrow O_3 \quad \text{(R9)}$$
$$Cl + O_3 \rightarrow ClO \quad \text{(R10)}$$
$$O_3 + h\nu \rightarrow O^1D + O_2 \quad \text{(R11)}$$
$$O^1D + H_2O \rightarrow 2OH \quad \text{(R12)}$$
$$ClO + HO_2 \rightarrow HOCl \quad \text{(R13)}$$
$$HOCl + h\nu \rightarrow Cl + OH \quad \text{(R14)}$$

Other reactive chlorine forms (e.g., HOCl and Cl_2) also play a role in the overall effects of BB-Cl on atmospheric chemistry. However, the activation of chlorine from relatively inert forms (such as SSA and HCl) predominantly occurs through the N_2O_5 heterogeneous reaction. Following this initial activation, gaseous chlorine species undergo recycling among several key species, including Cl_2 and HOCl, before deposition terminates the chlorine cycling process.

The CAM-Chem model used in our study does not specifically account for chloride in the aerosol chemical scheme; therefore, we have combined the emission of HCl and chloride together under the category "HCl" in the model simulation. Considering that (1) the main contribution of BB chlorine to the atmospheric chemistry over Asia is through $ClNO_2$, which is a product of N_2O_5 with chloride [42] or HCl in the presence of other aerosols [41], and (2) HCl and chloride co-exist in thermodynamic equilibrium, our assumption should be acceptable in reproducing real atmospheric conditions.

4 CAM-Chem model simulation and validation

By employing the CAM-Chem model, we conducted two main cases with (BB case) and without (noBB case) considering the newly compiled long-term BB-Cl emission inventory in the present study (Table S4). The difference in global atmospheric compositions between the noBB and BB cases represents the impact of BB Cl emissions from Asia. The rest of the model setups are identical for both cases: (1) For the sources of greenhouse gases (e.g., CH_4, long-lived chlorinated species, etc.), we followed the previous method used in Chemistry-Climate Model Initiative (CCMI) [33] to set up the lower boundary conditions (LBC) in CAM-Chem; an exception is that CH_3Cl LBC is excluded in both BB and noBB cases. (2) For the emissions of the routine atmospheric pollutants (CO, NO_x, VOCs, SO_2, NH_3, etc.), we utilized updated global emission inventories from the Coupled Model Intercomparison Project Phase 6 (CMIP6; for the years 1999-2015) [43] and shared socioeconomic pathways (SSP) 245 (SSP245; for the years 2016-2018) [44] for both the noBB and BB cases. (3) We adopted the specified dynamic mode (fixed meteorological files from the modern-era retrospective analysis for research and applications, version 2, MERRA2) of CAM-Chem to isolate the effects of BB Cl on the atmospheric composition and oxidation capacity. (4) The simulations were conducted globally with a horizontal resolution of approximately 1 degree, and 56 vertical layers. While the study encompasses global simulations, the results primarily focus on regions significantly impacted by Asian BB-Cl. (5) The simulations were conducted from 1999 to 2018, with the first two years considered as spin-up and thus discarded. The monthly average results of the remaining years (2001 to 2018) are presented in this study.

Furthermore, we conducted two additional short-period simulations from 2012 to 2014, with (BB_ANT) and without (noBB_ANT) the new BB-Cl emission inventory (Table S4), and both of them included anthropogenic emissions of reactive chlorine in China for 2014 [45]. The simulation results from the BB_ANT case in 2014 were used to evaluate the performance of the CAM-Chem model and the contributions of BB Cl to the overall atmospheric abundance and chemistry. We first compared the modelled O_3 and NO_2 with the observed mixing ratios (Fig. S3 and 4), and the results show that the global CAM-Chem model is able to reproduce the spatial distribution, seasonal variation, and magnitude of the surface O_3 and NO_2 levels in our domain of interest. The simulated HCHO levels in Asia are generally in line with the satellite-derived mixing ratios, although with some overestimation (Fig. S5). The magnitude of the simulated $ClNO_2$ (a critical chlorine species) mixing ratios in China was also reasonably reproduced by our global model (Table S5). However, limited field reports exist regarding Cl emissions and ambient levels in South and Southeast Asia. Further field studies on ambient Cl are necessary to verify the effects of BB-derived Cl sources and their impacts. In our simulations, BB-Cl was emitted from the ground surface, irrespective of their nature of emission (agricultural fire or large-scale forest wildfires). It's worth noting that this study focuses on examining the long-term trends of BB-Cl emissions and their general effects on atmospheric chemistry. We did not intend to investigate the individual large-scale wildfire episode.

5 Future BB Cl emission projection

The projected BB Cl emissions in the 21st century are based on the existing estimate of future biomass burning activities. To characterize a spectrum of potential scenarios, we utilize the novel Shared Socioeconomic Pathways (SSP) framework, designed to encompass diverse future trajectories arising from the interplay of socioeconomic and technological developments [43]. Here, we adopt the latest compilation of biomass burning emissions within the SSPs,

which are also used for the ongoing ScenarioMIP experiment within the CMIP6, from the year 2015 to the end of the 21st century. The gridded emissions are publicly available at https://esgf-node.llnl.gov/projects/input4mips/.

To capture the potential ranges of future biomass burning activity levels and the associated BB Cl emissions, we considered three emission scenarios, i.e., SSP1-2.6, SSP2-4.5, and SSP5-8.5, which are generally regarded as low, medium, and high emission scenarios. Our developed BB Cl emission inventory was projected toward the end of the century with reference to the predicted emission intensity of BB particulate matter and CO in the five key regions. To provide historically consistent and spatially detailed emissions datasets, the linear relationships of Cl^-/BC, CH_3Cl/CO and HCl/CO for each grid during the period 2001-2018 were established for the major regions in Asia. After the removal of the outliers, the mean parameters of the corresponding linear equations for each region were used as the scaling parameters to develop the projected BB Cl emission inventory.

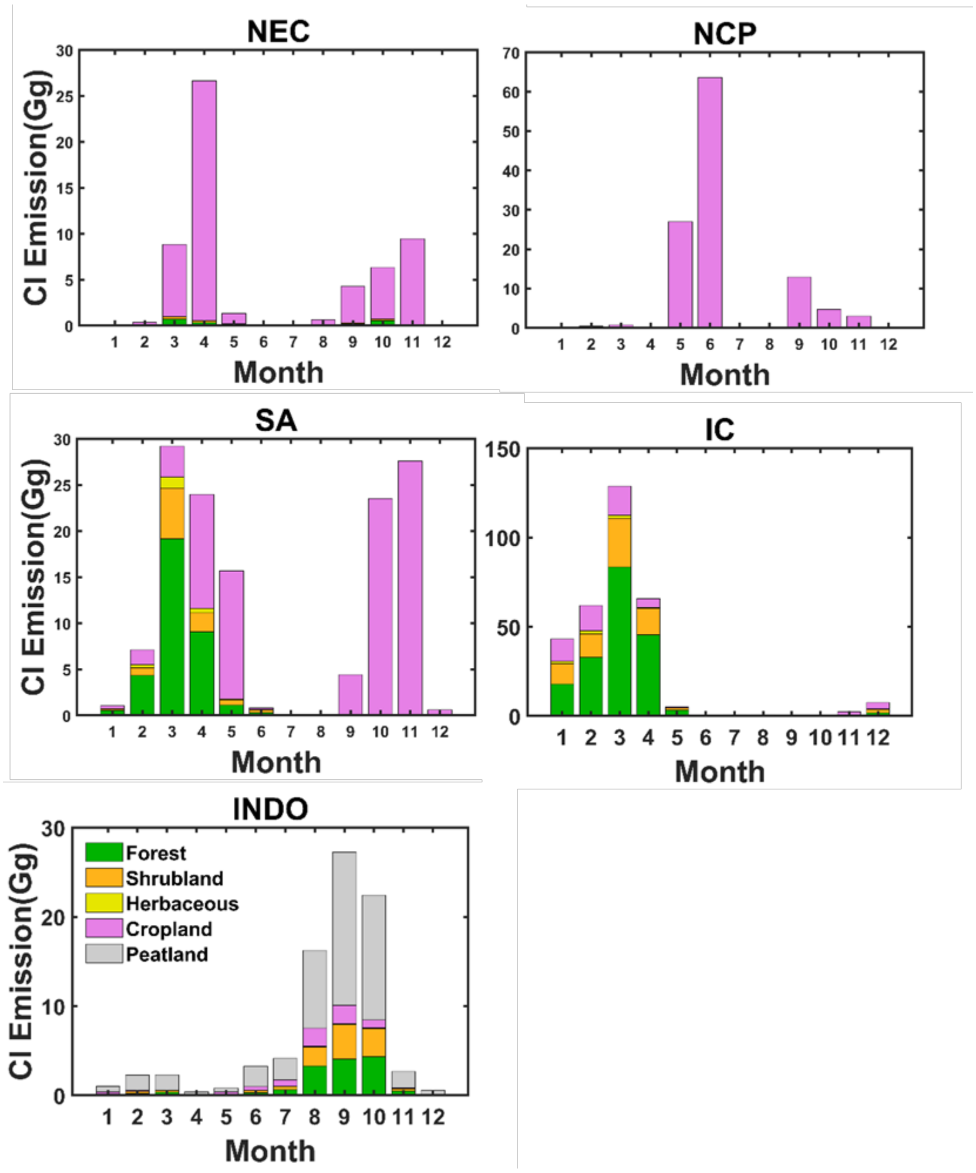

Fig. S1.

Monthly average chlorine (Cl) emissions from BB in the main regions in Asia over the period 2001-2018.

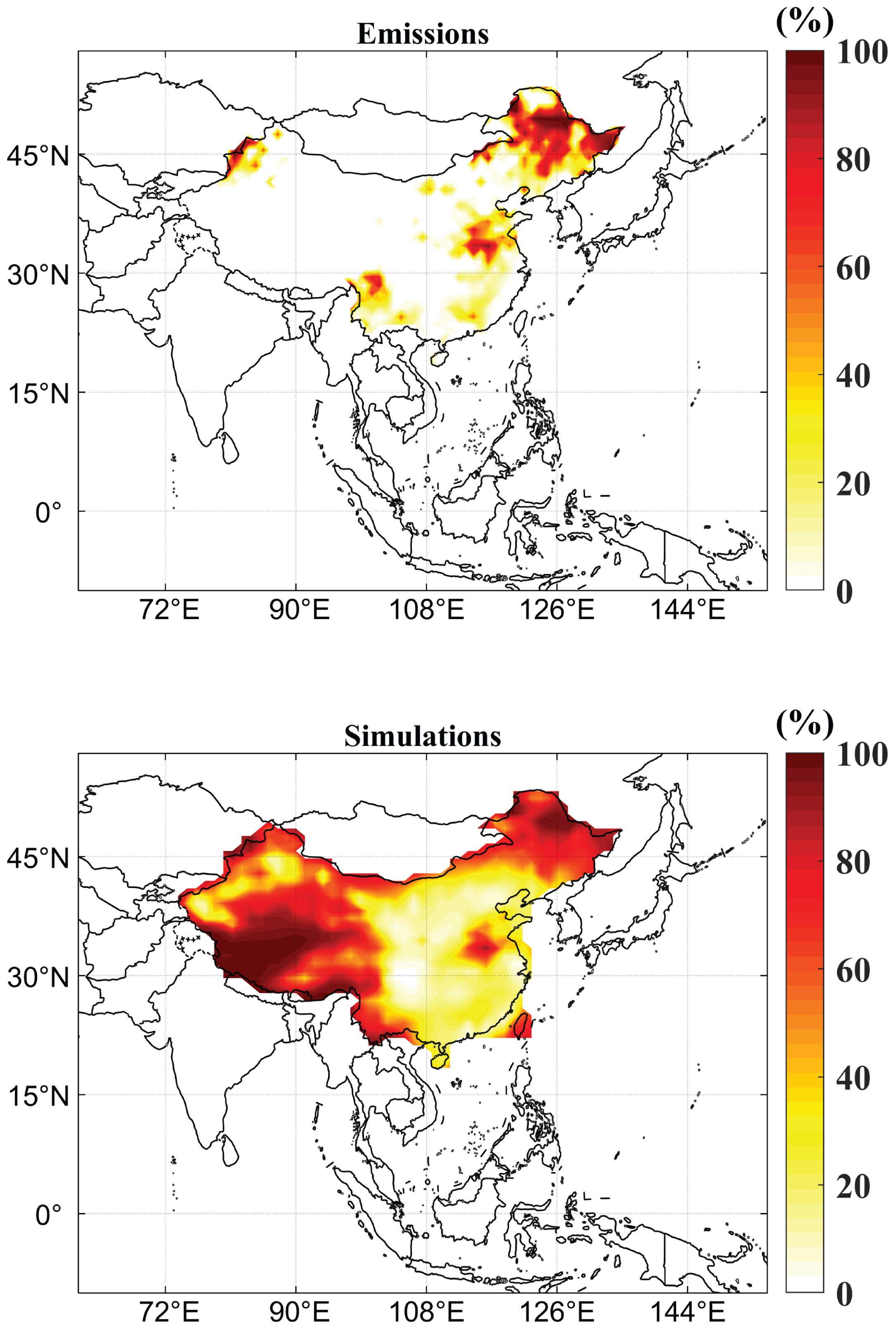

Fig. S2.

The contribution of BB to the total Cl emission inventory (top) and the BB's contribution to the total simulated Cl concentrations (bottom) for China for the year 2014. Note that the very high (close to 100%) BB contribution to the simulated Cl level in western China is due to the dispersion of long-lived CH_3Cl from source regions to the remote areas.

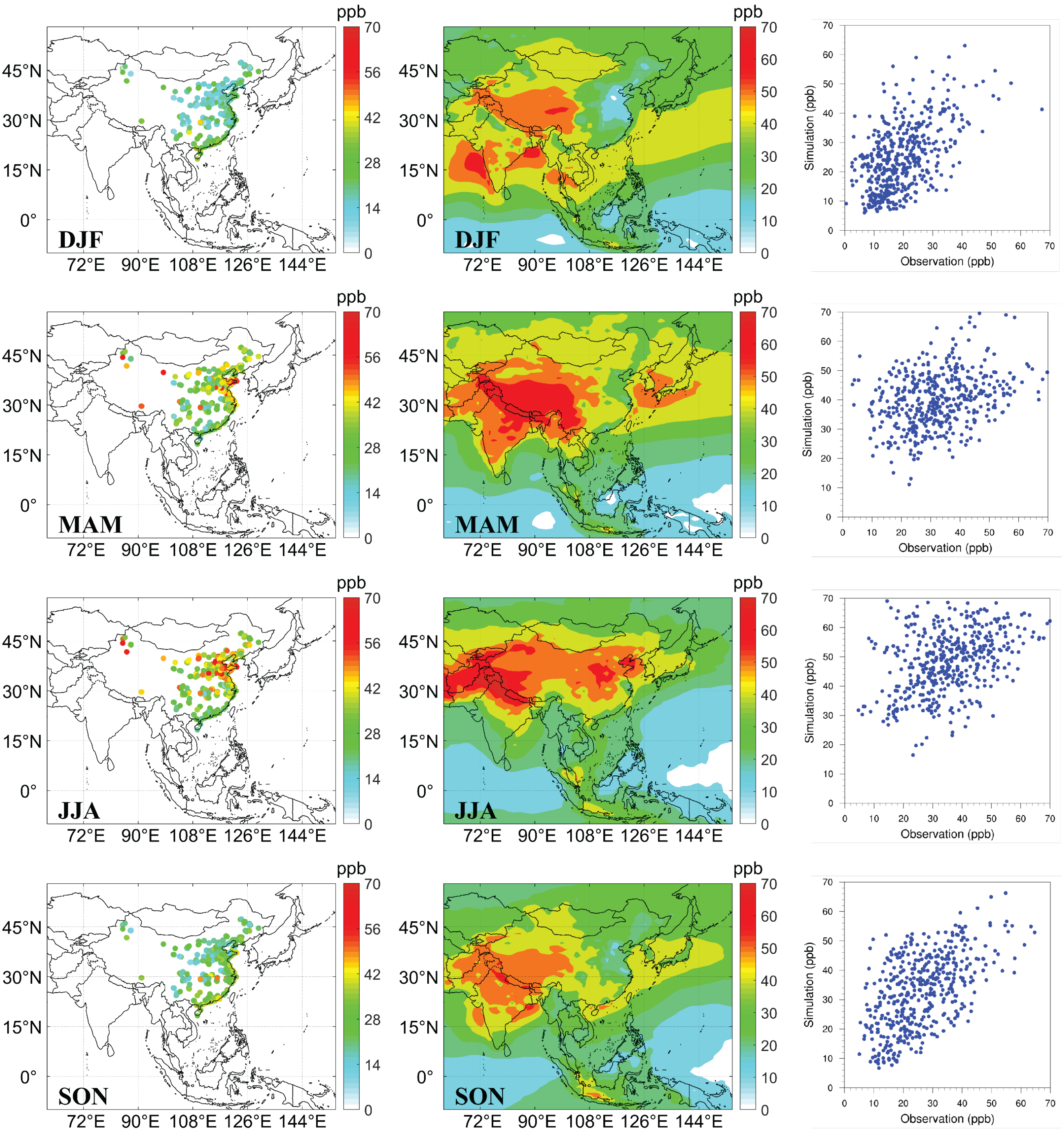

Fig. S3.

Seasonal comparison of ground surface O_3 concentrations between observations (left; https://www.mee.gov.cn/, last access: 2020/08/28) and model simulations (right; BB_ANT case) for the year 2014. The observed average O_3 mixing ratio are 32.7, 35.8, 26.2, and 19.6 ppbv in spring, summer, autumn, and winter, respectively, while simulation averages are 40.0, 48.4, 32.8, and 25.1 ppbv.

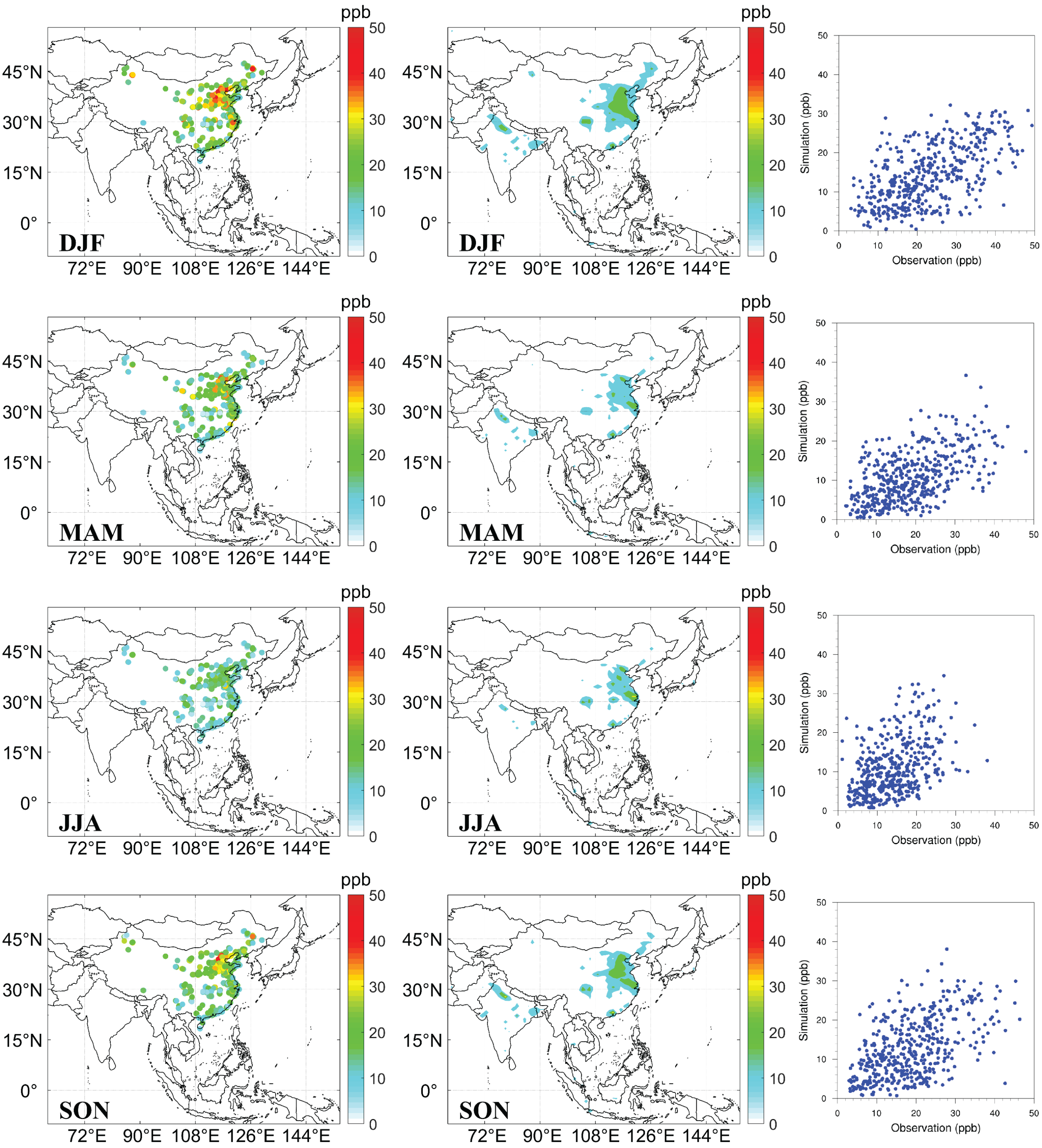

Fig. S4.

The same as Fig. S3 but for NO_2. Observation averages of NO_2 mixing ratio are 18.7, 13.9, 18.8, and 23.3 ppbv in spring, summer, autumn, and winter, respectively, and simulation averages are 10.6, 10.7, 12.8, and 14.4 ppbv.

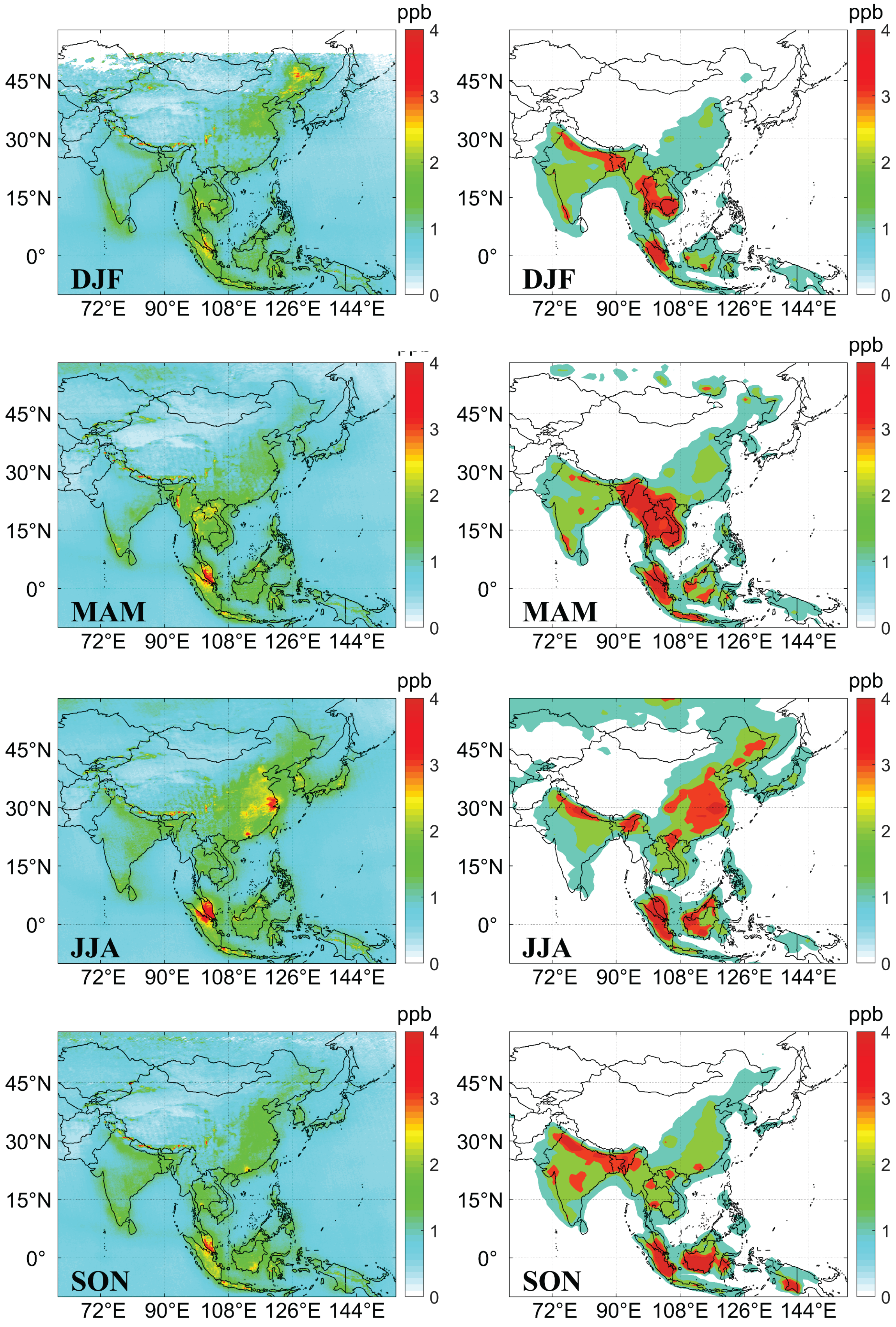

Fig. S5.

The same as Fig. S3 but with the satellite retrieval for HCHO (left).

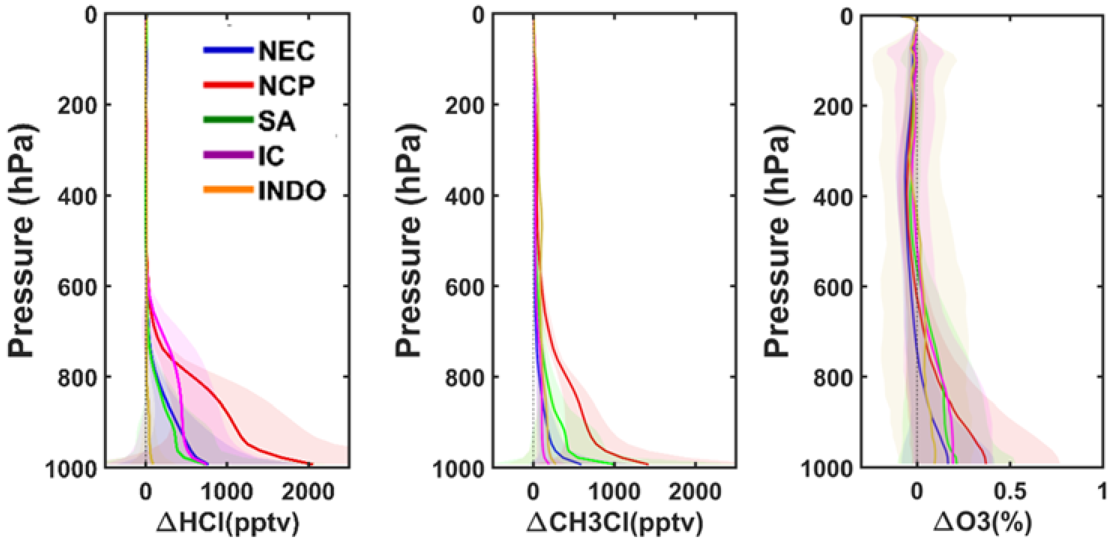

Fig. S6.
Simulated vertical profile of the change in HCl, CH_3Cl, and O_3 induced by BB Cl emissions.

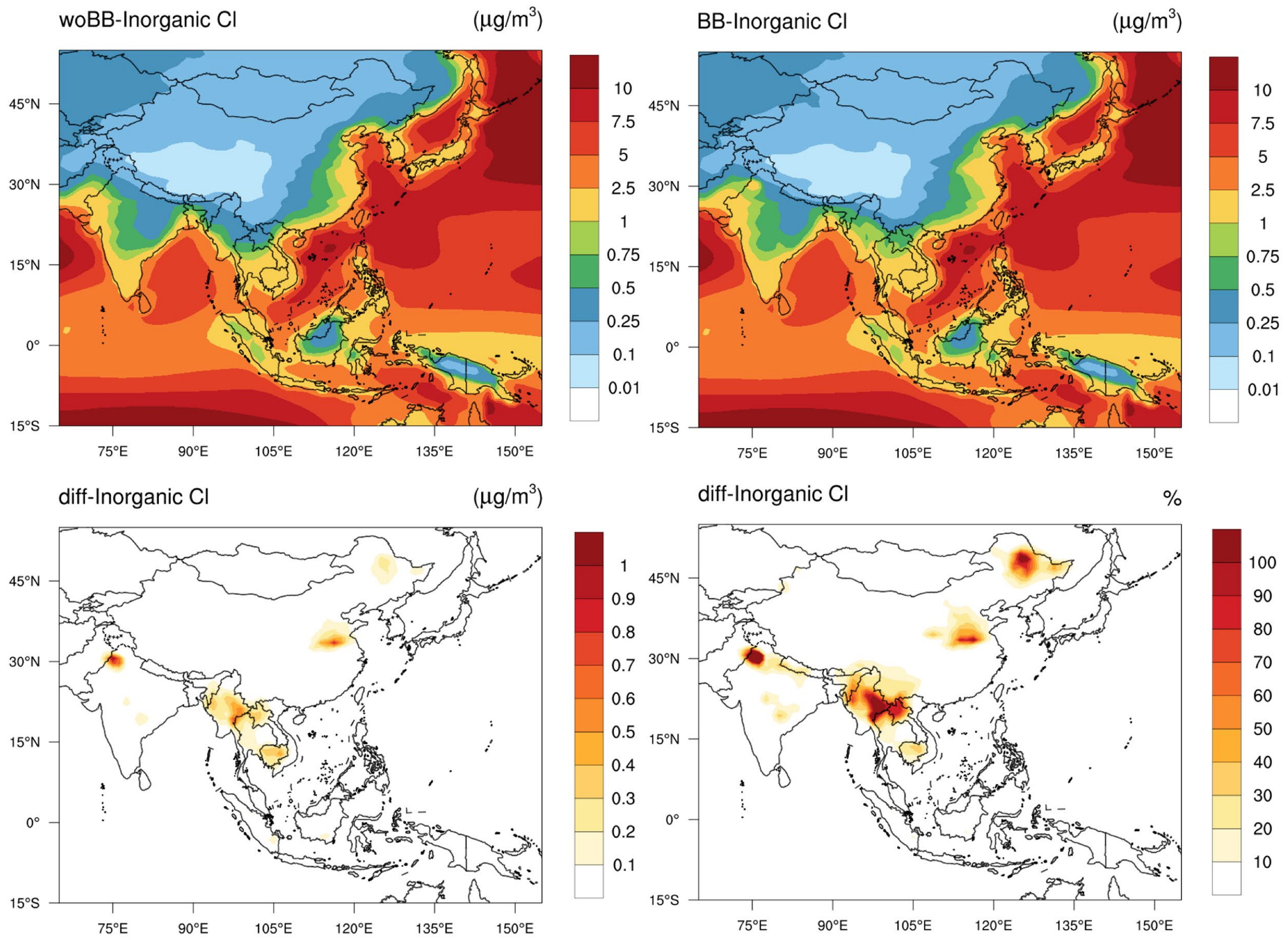

Fig. S7.
CAM-Chem simulated inorganic chlorine (gaseous and particulate inorganic chlorine) concentration (μg/m^3) in the woBB and BB cases, along with the absolute (μg/m^3) and (%) difference between the two cases.

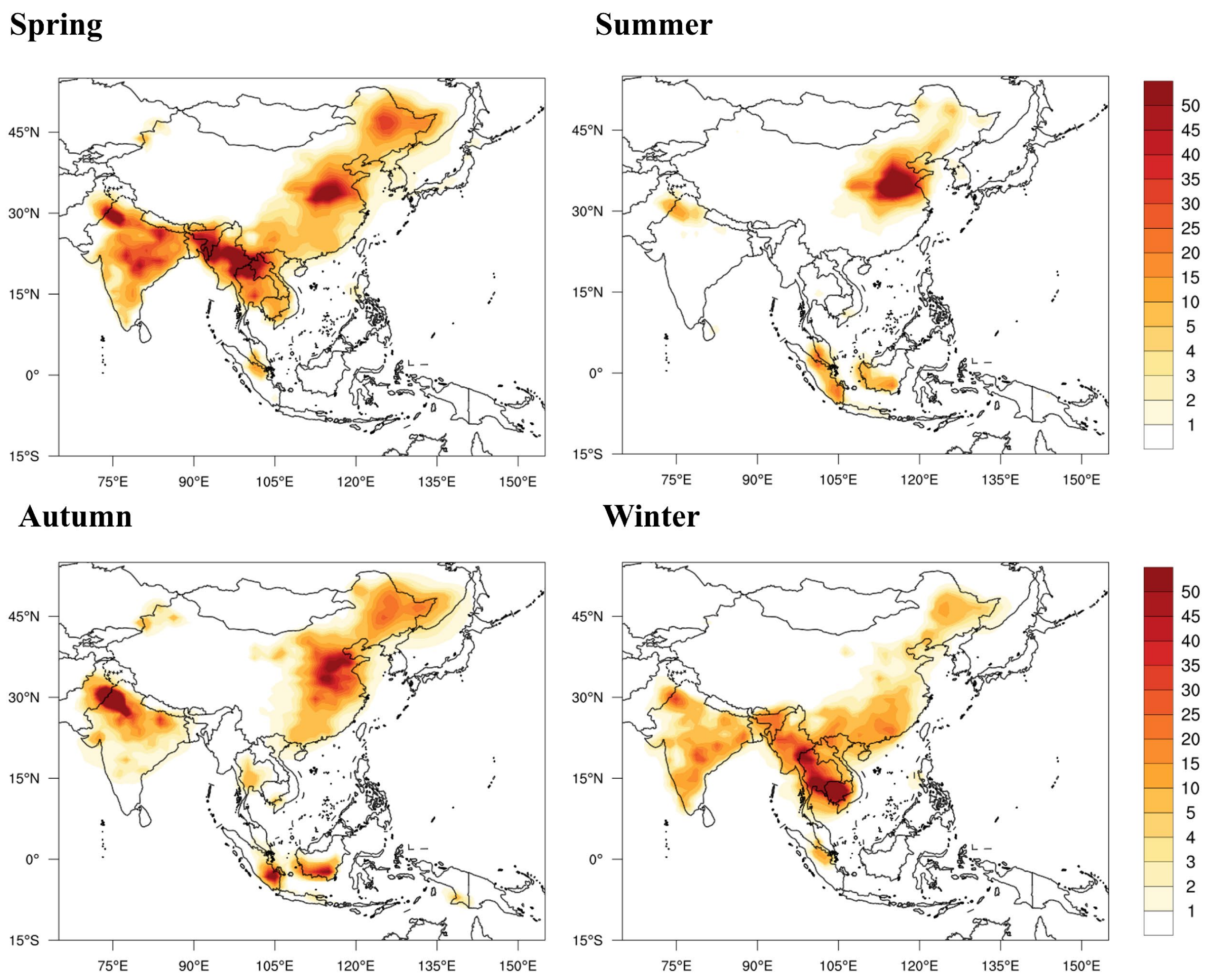

Fig. S8.

The seasonally averaged increase in the simulated $ClNO_2$ concentration (pptv) at the ground surface was attributed to BB Cl during 2001–2018.

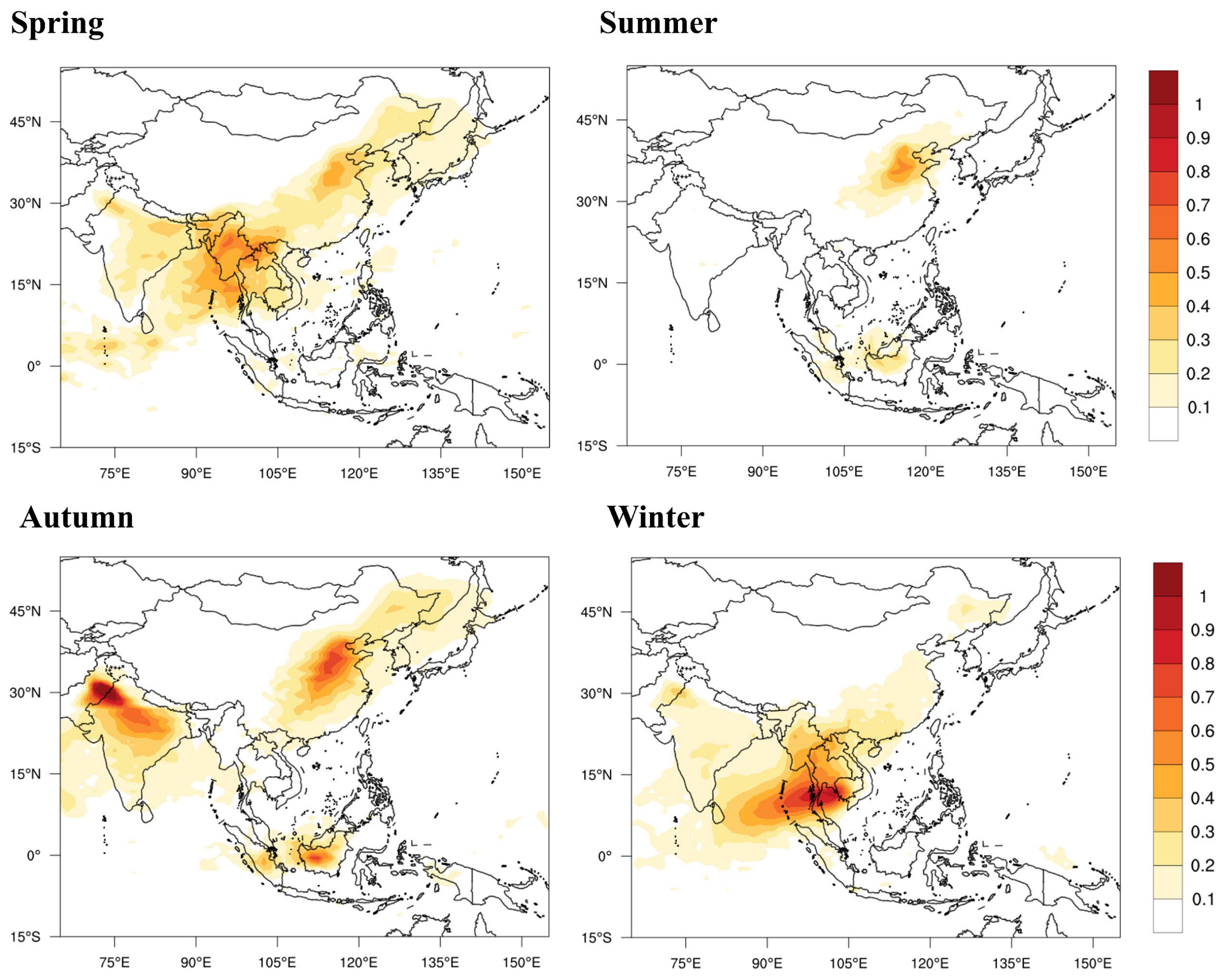

Fig. S9.

The same as Fig. S8 but for O_3 (%).

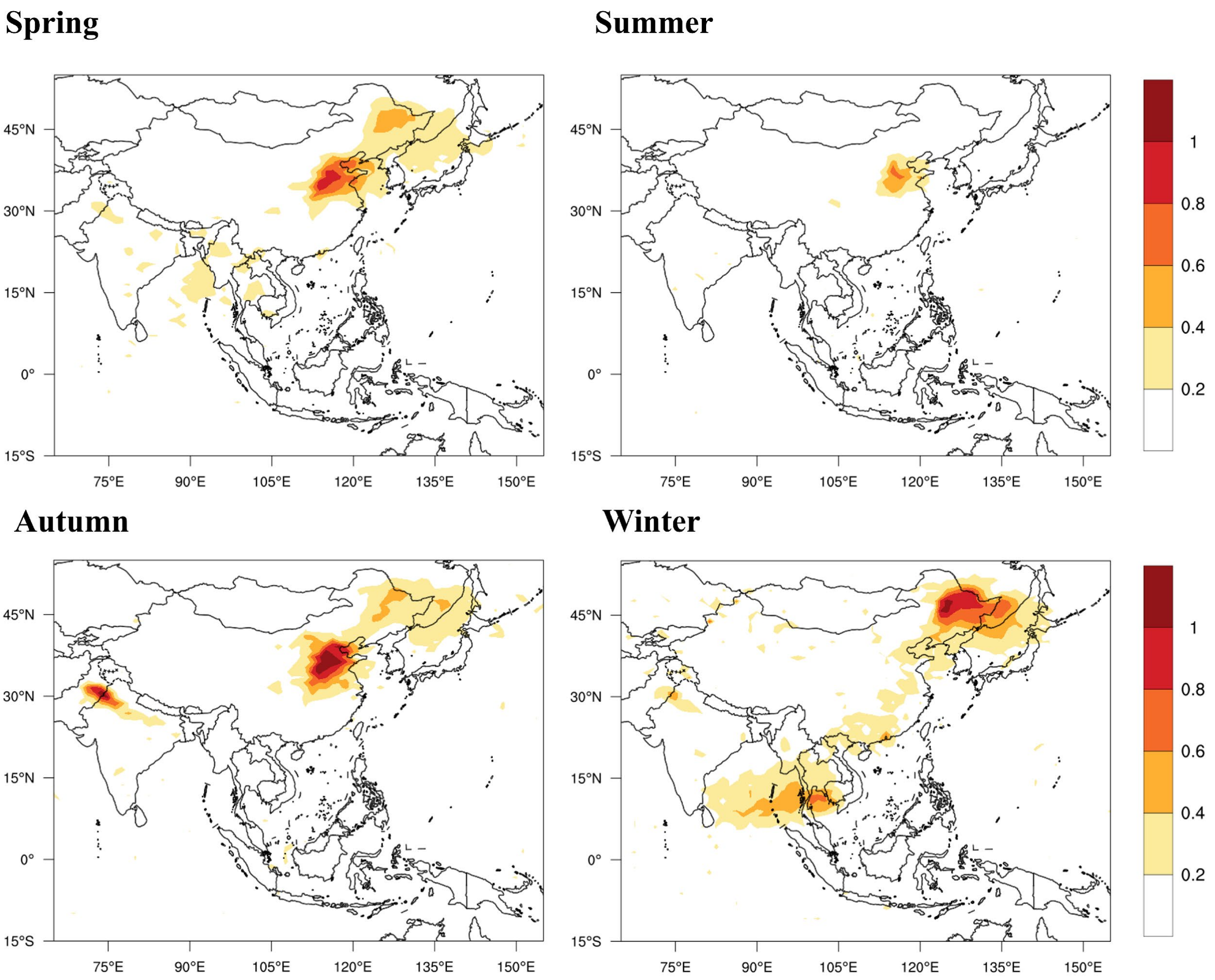

Fig. S10.

The same as Fig. S8 but for HO_x (%).

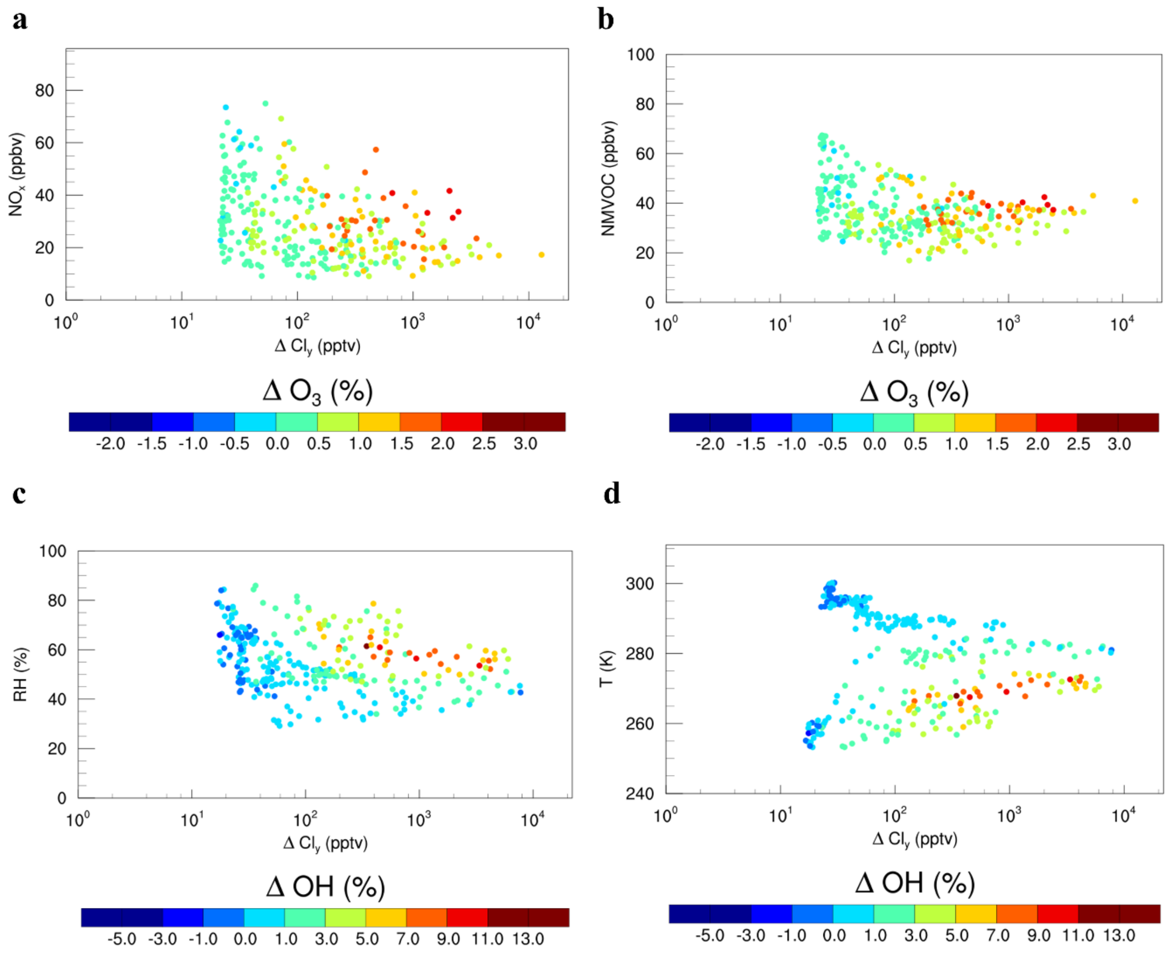

Fig. S11.

Connection between the NO_x level and O_3 change due to BB Cl emissions (a), VOC level and O_3 change (b), relative humidity and OH changes (c), and temperature and OH changes (d).

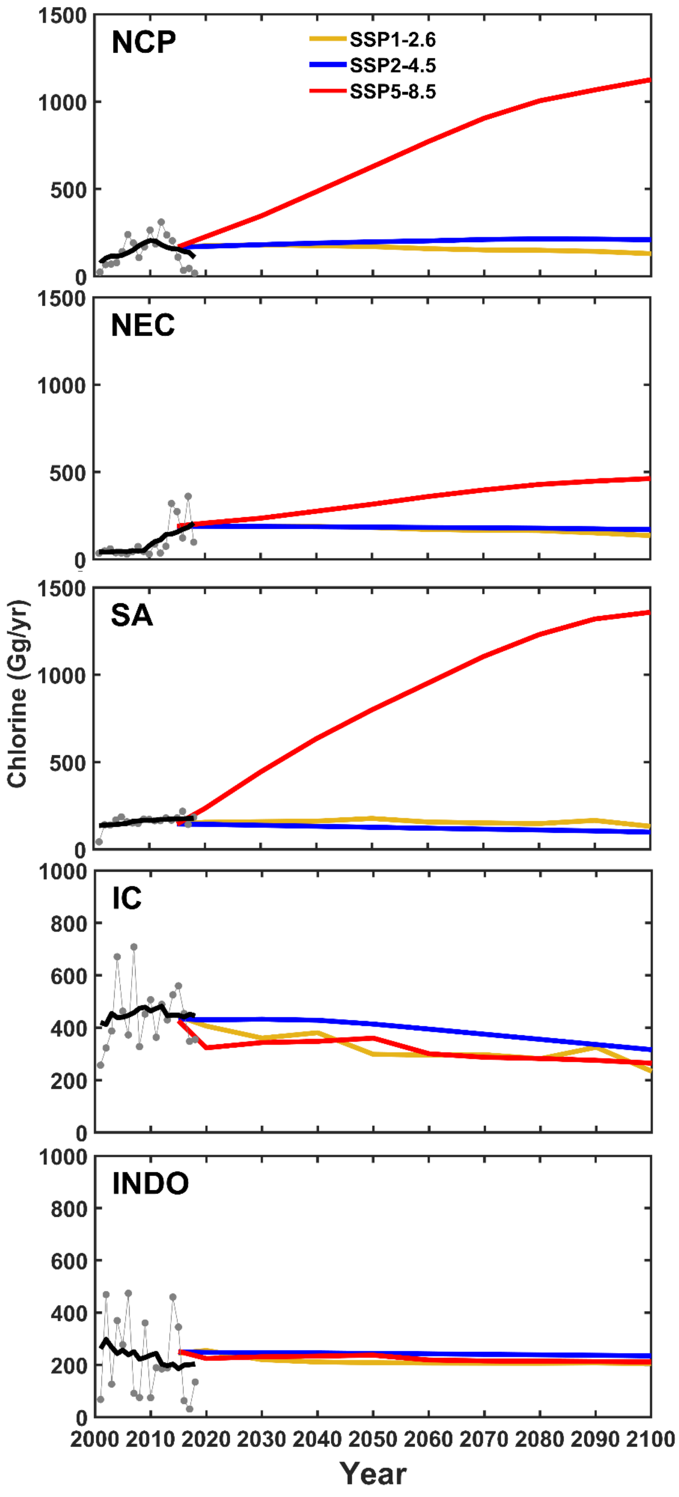

Fig. S12.

Average chlorine emissions for the current baseline (2001-2018, gray dots, black lines correspond to the moving average over a ten-year interval) and projection until 2100 under three SSP-based scenarios (SSP1-2.6, SSP2-4.5, SSP5-8.5) for the five selected regions.

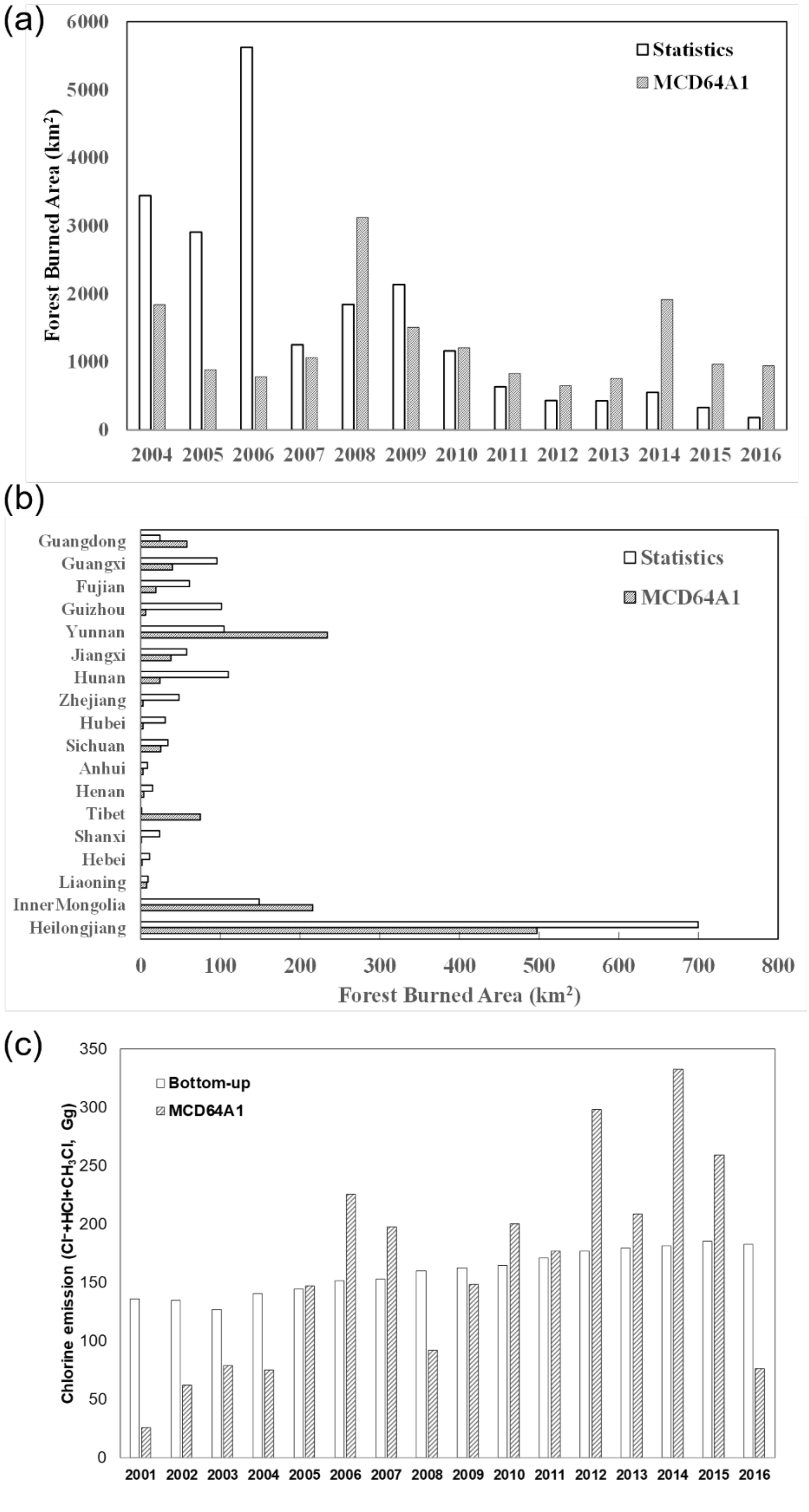

Fig. S13.

(a) Comparison between fire-affected forest area statistics and burned areas in forests derived from the MCD64A1 product in China over the period 2004−2016. (b) Comparison of annual average fire-affected forest area statistics with MCD64A1 results for major provinces in China. (c) Comparison of the total chlorine (Cl^{-}+HCl+CH_3Cl) emissions estimated by the bottom-up method and emissions from the MCD64A1 product for China during 2001−2016.

Table S1.

Annual chlorine emissions for the major countries in Asia (Unit: Gg).

Country	China	India	Cambodia	Thailand	Laos	Myanmar	Vietnam	Indonesia	Total
2001	33	32	34	37	5	67	6	11	240
2002	68	66	61	39	23	73	11	188	547
2003	104	97	67	40	25	123	29	49	558
2004	94	139	82	76	110	245	33	163	975
2005	158	110	63	69	55	146	43	64	735
2006	233	119	58	32	23	109	16	205	825
2007	211	133	81	82	122	274	40	18	979
2008	119	109	60	35	17	81	23	13	470
2009	166	187	70	48	28	167	25	132	847
2010	219	166	48	41	94	186	46	8	833
2011	185	136	67	27	16	101	20	58	629
2012	305	181	57	54	37	144	37	70	903
2013	217	154	52	35	54	128	39	48	751
2014	347	162	50	57	39	193	44	150	1083
2015	267	138	69	48	39	166	51	178	986
2016	84	160	50	51	63	93	42	16	581
2017	288	158	50	38	18	87	18	8	673
2018	85	165	56	28	19	79	26	55	534
Average	177	134	60	47	44	137	31	80	730

Table S2.

Comparisons of the BB Cl emissions from this work with previous chlorine estimates.

	RCEI[a]	Fu et al. [45][b]	Zhang et al. [46][b]	This work [b]
	Asia			
HCl			—	264
Cl^-	2526		55	311
	China			
HCl		146	—	110
Cl^-	937	364	43	171

[a] Representative for the year 1990.

[b] Representative for the year 2014.

Table S3.

Emission factors for Cl^-, HCl, and CH_3Cl for different vegetation types (unit: g kg^{-1})

Species	Forests	Shrublands	Grasslands	Crop Residue
Cl^-	0.54[22, 23]	0.43[22, 23]	0.23[47]	0.78[20]
HCl	0.41[24]	0.26[24]	0.06[24]	0.50[24]
CH_3Cl	0.059[47]	0.057[47]	0.075[48, 49]	0.31[21]

Table S4.

The setting of the CAM-Chem simulation scenarios.

Cases	Biomass burning emission of HCl and CH_3Cl	Anthropogenic emission of HCl	Source of routine species	Simulation period
noBB	No	No	Yes	1999-2018
BB	Yes	No	Yes	1999-2018
noBB_ANT	No	Yes	Yes	2012-2014
BB_ANT	Yes	Yes	Yes	2012-2014

Table S5.

Comparison of simulated $ClNO_2$ levels from the BB_ANT and noBB_ANT cases in 2014 with the observational data in China (unit: pptv).

Site	Observation period	Observation	BB_ANT		noBB_ANT	
			Range of monthly average	Mean value for corresponding Month	Range of monthly average	Mean value for corresponding Month
Hok Tsui	Aug 2012 [50]	148.0	11.3 to 135.6	11.8	11.3 to 127.6	11.8
	Oct 2018 [51]	163.5	11.3 to 135.6	73.2	11.3 to 127.6	71.4
	Nov-Dec 2018 [51]	210.6	11.3 to 135.6	76.1	11.3 to 127.6	74.2
Mt. TMS	Nov-Dec 2013 [52]	74.6 (nighttime)	11.3 to 135.6	76.1	11.3 to 127.6	74.2
Wangdu	Jun-Jul 2014 [53]	159.5	35.1 to 189.0	147.0	33.5 to 165.5	135.7
	Dec 2017 [54]	71.2	35.1 to 189.0	37.4	33.5 to 165.5	36.7
Mt. Tai	Jul-Aug 2014 [55]	54	50.1 to 275.7	177.2	49.2 to 167.0	142.9
	Mar-Apr 2018 [54]	179.1	50.1 to 275.7	133.8	49.2 to 167.0	128.7
Jinan	Aug-Sep 2014 [56]	94.0	50.1 to 275.7	246.0	49.2 to 167.0	164.0
Beijing	Apr-May 2017 [57]	220.1	20.3 to 125.7	93.6	19.9 to 110.3	87.6
	Jun 2017 [58]	174.3	20.3 to 125.7	83.7	19.9 to 110.3	80.3
	Jan-Feb 2018 [54]	75.8	20.3 to 125.7	54.6	19.9 to 110.3	53.9
Nanjing	Apr 2018 [59]	631.8	49.8 to 114.5	87.6	47.4 to 98.1	83.6

Table S6.

Sources of fuel load data for individual countries used in emission estimation.

Country	References Forest	Shrubland	Grassland	Cropland
China	[6, 9]	[7, 10, 11]	[8, 9]	[14, 15]
India	[60]	[61]	[61]	[16-18]
Korea, DPR	[62, 63]	[8]	[8]	[16-18]
Korea, Republic of	[62, 63]	[8]	[8]	[16-18]
Nepal	[64]	[64]	[64]	[16-18]
Brunei	[65]	[66]	[66]	[16-18]
Indonesia	[65]	[66]	[66]	[16-18]
Malaysia	[65]	[66]	[66]	[16-18]
Timor Leste	[65]	[66]	[66]	[16-18]
Other regions	[63, 65, 67]	[68]	[68]	[16-18]

References for Supplementary Materials

1. Seiler, W, Crutzen, PJ. Estimates of Gross and Net Fluxes of Carbon between the Biosphere and the Atmosphere from Biomass Burning. *Climatic Change*. 1980; **2**(3): 207-247.
2. Friedl, MA, Sulla-Menashe, D, Tan, B, *et al.* MODIS Collection 5 global land cover: Algorithm refinements and characterization of new datasets. *Remote Sensing of Environment*. 2010; **114**(1): 168-182.
3. Giglio, L, Boschetti, L, Roy, DP, *et al.* The Collection 6 MODIS burned area mapping algorithm and product. *Remote Sens Environ*. 2018; **217**: 72-85.
4. Hall, JV, Loboda, TV, Giglio, L, *et al.* A MODIS-based burned area assessment for Russian croplands: Mapping requirements and challenges. *Remote Sensing of Environment*. 2016; **184**: 506-521.
5. Zhu, CM, Kobayashi, H, Kanaya, Y, *et al.* Size-dependent validation of MODIS MCD64A1 burned area over six vegetation types in boreal Eurasia: Large underestimation in croplands. *Sci Rep-Uk*. 2017; **7**, 4181.
6. Su, YJ, Guo, QH, Xue, BL, *et al.* Spatial distribution of forest aboveground biomass in China: Estimation through combination of spaceborne lidar, optical imagery, and forest inventory data. *Remote Sensing of Environment*. 2016; **173**: 187-199.
7. Hu Hui-Feng, WZ-HLIUG-HFUB-J. VEGETATION CARBON STORAGE OF MAJOR SHRUBLANDS IN CHINA. *Chinese Journal of Plant Ecology*. 2006; **30**(4): 539-544.
8. Piao, SL, Fang, JY, He, J-S, *et al.* Spatial distribution of grassland biomass in China. *Acta Phytoecologica Sinica*. 2004; **28**: 491-498.
9. Wen, D, He, N. Spatial patterns of litter density and their controlling factors in forests and grasslands of China. 2016; **36**: 2876-2884.
10. Jie-Lin Ge, G-MXJ-XLIW-TXUC-MZZ-JLUY-LLIZ-Q. Litter standing crop of shrubland ecosystems in southern China. *Chinese Journal of Plant Ecology*. 2017; **41**(1): 5-13.
11. Xian Yang, Y-PGUOAMH-YLIUW-HMAS-LYUZ-YT. Distribution of biomass in relation to environments in shrublands of temperate China. *Chinese Journal of Plant Ecology*. 2017; **41**(1): 22-30.
12. Lobert, JM, Keene, WC, Logan, JA, *et al.* Global chlorine emissions from biomass burning: Reactive Chlorine Emissions Inventory. *J Geophys Res-Atmos*. 1999; **104**(D7): 8373-8389.
13. Yang, L, Wang, XY, Han, LP, *et al.* A quantitative assessment of crop residue feedstocks for biofuel in North and Northeast China. *Gcb Bioenergy*. 2015; **7**(1): 100-111.
14. (NBSC), NBoSoC. China Statistical Yearbook 2016. Beijing, China: China Statistics Press; 2017.
15. Wang, X, Xue, S, Xie, GH. Value-taking for residue factor as a parameter to assess the field residue of field crops. *Journal of China Agricultural University*. 2012; **17**(1): 1-8.
16. FAO. *FAO Statistical Yearbook 2004*. Rome: Food and Agriculture Organisation; 2006, 318 p.
17. Koopmans, A, Koppejan, J. Agricultural and forest residues-generation, utilization and availability. *Regional consultation on modern applications of biomass energy*. 1997; 6-10 January.
18. Yevich, R, Logan, JA. An assessment of biofuel use and burning of agricultural waste in the developing world. *Global Biogeochem Cy*. 2003; **17**(4): 1095.
19. Hoffa, EA, Ward, DE, Hao, WM, *et al.* Seasonality of carbon emissions from biomass burning in a Zambian savanna. *J Geophys Res-Atmos*. 1999; **104**(D11): 13841-13853.
20. Li, CL, Hu, YJ, Zhang, F, *et al.* Multi-pollutant emissions from the burning of major agricultural residues in China and the related health-economic effects. *Atmos Chem Phys*. 2017; **17**(8): 4957-4988.
21. Li, XH, Wang, SX, Hao, JM. Characteristics of volatile organic compounds (VOCs) emitted from biofuel combustion in China. *Huan Jing Ke Xue*. 2011; **32**(12): 3515-3521.
22. Liu, G, Huang, K, Li, J-H, *et al.* Chemical Composition of Water-soluble Ions in Smoke Emitted from Tree Branch Combustion. *Huan jing ke xue= Huanjing kexue / [bian ji, Zhongguo ke xue yuan huan jing ke xue wei yuan hui "Huan jing ke xue" bian ji wei yuan hui]*. 2016; **37**: 3737-3742.
23. Liu, G, Li, JH, Xu, H, *et al.* Chemical composition of water-soluble ions in smoke from leaf combustion. *Zhongguo Huanjing Kexue/China Environmental Science*. 2017; **37**: 4480-4486.
24. Stockwell, CE, Yokelson, RJ, Kreidenweis, SM, *et al.* Trace gas emissions from combustion of peat, crop residue, domestic biofuels, grasses, and other fuels: configuration and Fourier transform infrared (FTIR) component of the fourth Fire Lab at Missoula Experiment (FLAME-4). *Atmos Chem Phys*. 2014; **14**(18): 9727-9754.
25. Michel, C, Liousse, C, Gregoire, JM, *et al.* Biomass burning emission inventory from burnt area data given by the SPOT-VEGETATION system in the frame of TRACE-P and ACE-Asia campaigns. *J Geophys Res-Atmos*. 2005; **110**(D9), D09304.
26. Ito, A, Penner, JE. Global estimates of biomass burning emissions based on satellite imagery for the year 2000. *J Geophys Res-Atmos*. 2004; **109**(D14), D14S05.

27. Li, J, Bo, Y, Xie, SD. Estimating emissions from crop residue open burning in China based on statistics and MODIS fire products. *J Environ Sci-China*. 2016; **44**: 158-170.
28. Roy, DP, Boschetti, L, Justice, CO*, et al.* The collection 5 MODIS burned area product - Global evaluation by comparison with the MODIS active fire product. *Remote Sensing of Environment*. 2008; **112**(9): 3690-3707.
29. Zhou, Y, Xing, XF, Lang, JL*, et al.* A comprehensive biomass burning emission inventory with high spatial and temporal resolution in China. *Atmos Chem Phys*. 2017; **17**(4): 2839-2864.
30. Peng, L, Zhang, Q, He, K. Emissions inventory of atmospheric pollutants from open burning of crop residues in China based on a national questionnaire. *Research of Environmental Sciences*. 2016; **29**: 1109-1118.
31. Wu, J, Kong, SF, Wu, FQ*, et al.* Estimating the open biomass burning emissions in central and eastern China from 2003 to 2015 based on satellite observation. *Atmos Chem Phys*. 2018; **18**(16): 11623-11646.
32. Lamarque, JF, Emmons, LK, Hess, PG*, et al.* CAM-chem: description and evaluation of interactive atmospheric chemistry in the Community Earth System Model. *Geosci Model Dev*. 2012; **5**(2): 369-411.
33. Tilmes, S, Lamarque, JF, Emmons, LK*, et al.* Representation of the Community Earth System Model (CESM1) CAM4-chem within the Chemistry-Climate Model Initiative (CCMI). *Geosci Model Dev*. 2016; **9**(5): 1853-1890.
34. Iglesias-Suarez, F, Badia, A, Fernandez, RP*, et al.* Natural halogens buffer tropospheric ozone in a changing climate. *Nat Clim Change*. 2020; **10**(2): 147–154.
35. Cuevas, CA, Maffezzoli, N, Corella, JP*, et al.* Rapid increase in atmospheric iodine levels in the North Atlantic since the mid-20th century. *Nature Communications*. 2018; **9**, 1452.
36. Fernandez, RP, Salawitch, RJ, Kinnison, DE*, et al.* Bromine partitioning in the tropical tropopause layer: implications for stratospheric injection. *Atmos Chem Phys*. 2014; **14**(24): 13391-13410.
37. Ordonez, C, Lamarque, JF, Tilmes, S*, et al.* Bromine and iodine chemistry in a global chemistry-climate model: description and evaluation of very short-lived oceanic sources. *Atmos Chem Phys*. 2012; **12**(3): 1423-1447.
38. Saiz-Lopez, A, Fernandez, RP, Ordonez, C*, et al.* Iodine chemistry in the troposphere and its effect on ozone. *Atmos Chem Phys*. 2014; **14**(23): 13119-13143.
39. Simpson, WR, Brown, SS, Saiz-Lopez, A*, et al.* Tropospheric Halogen Chemistry: Sources, Cycling, and Impacts. *Chem Rev*. 2015; **115**(10): 4035-4062.
40. Saiz-Lopez, A, von Glasow, R. Reactive halogen chemistry in the troposphere. *Chem Soc Rev*. 2012; **41**(19): 6448-6472.
41. Raff, JD, Njegic, B, Chang, WL*, et al.* Chlorine activation indoors and outdoors via surface-mediated reactions of nitrogen oxides with hydrogen chloride. *Proc Natl Acad Sci U S A*. 2009; **106**(33): 13647-13654.
42. Thornton, JA, Kercher, JP, Riedel, TP*, et al.* A large atomic chlorine source inferred from mid-continental reactive nitrogen chemistry. *Nature*. 2010; **464**(7286): 271-274.
43. Gidden, MJ, Riahi, K, Smith, SJ*, et al.* Global emissions pathways under different socioeconomic scenarios for use in CMIP6: a dataset of harmonized emissions trajectories through the end of the century. *Geosci Model Dev*. 2019; **12**(4): 1443-1475.
44. Turnock, ST, Allen, RJ, Andrews, M*, et al.* Historical and future changes in air pollutants from CMIP6 models. *Atmos Chem Phys*. 2020; **20**(23): 14547-14579.
45. Fu, X, Wang, T, Wang, SX*, et al.* Anthropogenic Emissions of Hydrogen Chloride and Fine Particulate Chloride in China. *Environ Sci Technol*. 2018; **52**(3): 1644-1654.
46. Zhang, B, Shen, H, Yun, X*, et al.* Global Emissions of Hydrogen Chloride and Particulate Chloride from Continental Sources. *Environ Sci Technol*. 2022; **56**(7): 3894-3904.
47. Akagi, SK, Yokelson, RJ, Wiedinmyer, C*, et al.* Emission factors for open and domestic biomass burning for use in atmospheric models. *Atmos Chem Phys*. 2011; **11**(9): 4039-4072.
48. Andreae, MO. Emission of trace gases and aerosols from biomass burning - an updated assessment. *Atmos Chem Phys*. 2019; **19**(13): 8523-8546.
49. Engel, A, Rigby, M, Burkholder, JB*, et al.* Update on ozone-depleting substances (ODSs) and other gases of interest to the Montreal Protocol. In: Doherty, SJe, Means, Te, Stewart, BCe, et al. (eds.). *Scientific assessment of ozone depletion: 2018*. Geneva: WMO (World Meteorological Organization); 2019.
50. Tham, YJ, Yan, C, Xue, LK*, et al.* Presence of high nitryl chloride in Asian coastal environment and its impact on atmospheric photochemistry. *Chinese Sci Bull*. 2014; **59**(4): 356-359.
51. Peng, X, Wang, T, Wang, W*, et al.* Photodissociation of particulate nitrate as a source of daytime tropospheric Cl2. *Nature Communications*. 2022; **13**(1), 939.
52. Wang, T, Tham, YJ, Xue, LK*, et al.* Observations of nitryl chloride and modeling its source and effect on ozone in the planetary boundary layer of southern China. *J Geophys Res-Atmos*. 2016; **121**(5): 2476-2489.

53. Tham, YJ, Wang, Z, Li, QY, *et al.* Significant concentrations of nitryl chloride sustained in the morning: investigations of the causes and impacts on ozone production in a polluted region of northern China. *Atmos Chem Phys*. 2016; **16**(23): 14959-14977.
54. Xia, M, Peng, X, Wang, WH, *et al.* Winter ClNO2 formation in the region of fresh anthropogenic emissions: seasonal variability and insights into daytime peaks in northern China. *Atmos Chem Phys*. 2021; **21**(20): 15985-16000.
55. Wang, Z, Wang, WH, Tham, YJ, *et al.* Fast heterogeneous N2O5 uptake and ClNO2 production in power plant and industrial plumes observed in the nocturnal residual layer over the North China Plain. *Atmos Chem Phys*. 2017; **17**(20): 12361-12378.
56. Wang, XF, Wang, H, Xue, LK, *et al.* Observations of N2O5 and ClNO2 at a polluted urban surface site in North China: High N2O5 uptake coefficients and low ClNO2 product yields. *Atmos Environ*. 2017; **156**: 125-134.
57. Xia, M, Wang, WH, Wang, Z, *et al.* Heterogeneous Uptake of N2O5 in Sand Dust and Urban Aerosols Observed during the Dry Season in Beijing. *Atmosphere-Basel*. 2019; **10**(4), 204.
58. Zhou, W, Zhao, J, Ouyang, B, *et al.* Production of N2O5 and ClNO2 in summer in urban Beijing, China. *Atmos Chem Phys*. 2018; **18**(16): 11581-11597.
59. Xia, M, Peng, X, Wang, WH, *et al.* Significant production of ClNO2 and possible source of Cl-2 from N2O5 uptake at a suburban site in eastern China. *Atmos Chem Phys*. 2020; **20**(10): 6147-6158.
60. Chhabra, A, Dadhwal, VK. Assessment of major pools and fluxes of carbon in Indian forests. *Climatic Change*. 2004; **64**(3): 341-360.
61. Singh, JS, Yadava, PS. Seasonal-Variation in Composition, Plant Biomass, and Net Primary Productivity of a Tropical Grassland at Kurukshetra, India. *Ecol Monogr*. 1974; **44**(3): 351-376.
62. Choi, SD, Lee, K, Chang, YS. Large rate of uptake of atmospheric carbon dioxide by planted forest biomass in Korea. *Global Biogeochem Cy*. 2002; **16**(4), 1089.
63. FAO. *State of the World's Forests 2005*. Rome: Food and Agriculture Organisation; 2005, 166p.
64. Shrestha, BM, Singh, BR. Soil and vegetation carbon pools in a mountainous watershed of Nepal. *Nutr Cycl Agroecosys*. 2008; **81**(2): 179-191.
65. Brown, S, Iverson, LR, Prasad, A, *et al.* Geographical distributions of carbon in biomass and soils of tropical Asian forests. *Geocarto International*. 1993; **8**(4): 45-59.
66. Hashimotio, T, Kojima, K, Tange, T, *et al.* Changes in carbon storage in fallow forests in the tropical lowlands of Borneo. *Forest Ecol Manag*. 2000; **126**(3): 331-337.
67. Brown, S. *Estimating biomass and biomass change of tropical forests*: Food & Agriculture Org.; 1997, 134p.
68. Christian, TJ, Kleiss, B, Yokelson, RJ, *et al.* Comprehensive laboratory measurements of biomass-burning emissions: 1. Emissions from Indonesian, African, and other fuels. *J Geophys Res-Atmos*. 2003; **108**(D23), 4719.
